# Search for physics beyond the standard model in events with jets and two same-sign or at least three charged leptons in proton-proton collisions at $\sqrt{s}=13\,{\text {TeV}}$

**DOI:** 10.1140/epjc/s10052-020-8168-3

**Published:** 2020-08-18

**Authors:** A. M. Sirunyan, A. Tumasyan, W. Adam, F. Ambrogi, T. Bergauer, M. Dragicevic, J. Erö, A. Escalante Del Valle, M. Flechl, R. Frühwirth, M. Jeitler, N. Krammer, I. Krätschmer, D. Liko, T. Madlener, I. Mikulec, N. Rad, J. Schieck, R. Schöfbeck, M. Spanring, W. Waltenberger, C.-E. Wulz, M. Zarucki, V. Drugakov, V. Mossolov, J. Suarez Gonzalez, M. R. Darwish, E. A. De Wolf, D. Di Croce, X. Janssen, A. Lelek, M. Pieters, H. Rejeb Sfar, H. Van Haevermaet, P. Van Mechelen, S. Van Putte, N. Van Remortel, F. Blekman, E. S. Bols, S. S. Chhibra, J. D’Hondt, J. De Clercq, D. Lontkovskyi, S. Lowette, I. Marchesini, S. Moortgat, Q. Python, S. Tavernier, W. Van Doninck, P. Van Mulders, D. Beghin, B. Bilin, B. Clerbaux, G. De Lentdecker, H. Delannoy, B. Dorney, L. Favart, A. Grebenyuk, A. K. Kalsi, L. Moureaux, A. Popov, N. Postiau, E. Starling, L. Thomas, C. Vander Velde, P. Vanlaer, D. Vannerom, T. Cornelis, D. Dobur, I. Khvastunov, M. Niedziela, C. Roskas, K. Skovpen, M. Tytgat, W. Verbeke, B. Vermassen, M. Vit, O. Bondu, G. Bruno, C. Caputo, P. David, C. Delaere, M. Delcourt, A. Giammanco, V. Lemaitre, J. Prisciandaro, A. Saggio, M. Vidal Marono, P. Vischia, J. Zobec, G. A. Alves, G. Correia Silva, C. Hensel, A. Moraes, E. Belchior Batista Das Chagas, W. Carvalho, J. Chinellato, E. Coelho, E. M. Da Costa, G. G. Da Silveira, D. De Jesus Damiao, C. De Oliveira Martins, S. Fonseca De Souza, L. M. Huertas Guativa, H. Malbouisson, J. Martins, D. Matos Figueiredo, M. Medina Jaime, M. Melo De Almeida, C. Mora Herrera, L. Mundim, H. Nogima, W. L. Prado Da Silva, P. Rebello Teles, L. J. Sanchez Rosas, A. Santoro, A. Sznajder, M. Thiel, E. J. Tonelli Manganote, F. Torres Da Silva De Araujo, A. Vilela Pereira, C. A. Bernardes, L. Calligaris, T. R. Fernandez Perez Tomei, E. M. Gregores, D. S. Lemos, P. G. Mercadante, S. F. Novaes, S. S. Padula, A. Aleksandrov, G. Antchev, R. Hadjiiska, P. Iaydjiev, M. Misheva, M. Rodozov, M. Shopova, G. Sultanov, M. Bonchev, A. Dimitrov, T. Ivanov, L. Litov, B. Pavlov, P. Petkov, A. Petrov, W. Fang, X. Gao, L. Yuan, M. Ahmad, Z. Hu, Y. Wang, G. M. Chen, H. S. Chen, M. Chen, C. H. Jiang, D. Leggat, H. Liao, Z. Liu, A. Spiezia, J. Tao, E. Yazgan, H. Zhang, S. Zhang, J. Zhao, A. Agapitos, Y. Ban, G. Chen, A. Levin, J. Li, L. Li, Q. Li, Y. Mao, S. J. Qian, D. Wang, Q. Wang, M. Xiao, C. Avila, A. Cabrera, C. Florez, C. F. González Hernández, M. A. Segura Delgado, J. Mejia Guisao, J. D. Ruiz Alvarez, C. A. Salazar González, N. Vanegas Arbelaez, D. Giljanović, N. Godinovic, D. Lelas, I. Puljak, T. Sculac, Z. Antunovic, M. Kovac, V. Brigljevic, D. Ferencek, K. Kadija, B. Mesic, M. Roguljic, A. Starodumov, T. Susa, M. W. Ather, A. Attikis, E. Erodotou, A. Ioannou, M. Kolosova, S. Konstantinou, G. Mavromanolakis, J. Mousa, C. Nicolaou, F. Ptochos, P. A. Razis, H. Rykaczewski, H. Saka, D. Tsiakkouri, M. Finger, M. Finger, A. Kveton, J. Tomsa, E. Ayala, E. Carrera Jarrin, H. Abdalla, S. Khalil, S. Bhowmik, A. Carvalho Antunes De Oliveira, R. K. Dewanjee, K. Ehataht, M. Kadastik, M. Raidal, C. Veelken, P. Eerola, L. Forthomme, H. Kirschenmann, K. Osterberg, M. Voutilainen, F. Garcia, J. Havukainen, J. K. Heikkilä, V. Karimäki, M. S. Kim, R. Kinnunen, T. Lampén, K. Lassila-Perini, S. Laurila, S. Lehti, T. Lindén, H. Siikonen, E. Tuominen, J. Tuominiemi, P. Luukka, T. Tuuva, M. Besancon, F. Couderc, M. Dejardin, D. Denegri, B. Fabbro, J. L. Faure, F. Ferri, S. Ganjour, A. Givernaud, P. Gras, G. Hamel de Monchenault, P. Jarry, C. Leloup, B. Lenzi, E. Locci, J. Malcles, J. Rander, A. Rosowsky, M. Ö. Sahin, A. Savoy-Navarro, M. Titov, G. B. Yu, S. Ahuja, C. Amendola, F. Beaudette, P. Busson, C. Charlot, B. Diab, G. Falmagne, R. Granier de Cassagnac, I. Kucher, A. Lobanov, C. Martin Perez, M. Nguyen, C. Ochando, P. Paganini, J. Rembser, R. Salerno, J. B. Sauvan, Y. Sirois, A. Zabi, A. Zghiche, J.-L. Agram, J. Andrea, D. Bloch, G. Bourgatte, J.-M. Brom, E. C. Chabert, C. Collard, E. Conte, J.-C. Fontaine, D. Gelé, U. Goerlach, M. Jansová, A.-C. Le Bihan, N. Tonon, P. Van Hove, S. Gadrat, S. Beauceron, C. Bernet, G. Boudoul, C. Camen, A. Carle, N. Chanon, R. Chierici, D. Contardo, P. Depasse, H. El Mamouni, J. Fay, S. Gascon, M. Gouzevitch, B. Ille, Sa. Jain, I. B. Laktineh, H. Lattaud, A. Lesauvage, M. Lethuillier, L. Mirabito, S. Perries, V. Sordini, L. Torterotot, G. Touquet, M. Vander Donckt, S. Viret, A. Khvedelidze, Z. Tsamalaidze, C. Autermann, L. Feld, K. Klein, M. Lipinski, D. Meuser, A. Pauls, M. Preuten, M. P. Rauch, J. Schulz, M. Teroerde, M. Erdmann, B. Fischer, S. Ghosh, T. Hebbeker, K. Hoepfner, H. Keller, L. Mastrolorenzo, M. Merschmeyer, A. Meyer, P. Millet, G. Mocellin, S. Mondal, S. Mukherjee, D. Noll, A. Novak, T. Pook, A. Pozdnyakov, T. Quast, M. Radziej, Y. Rath, H. Reithler, J. Roemer, A. Schmidt, S. C. Schuler, A. Sharma, S. Wiedenbeck, S. Zaleski, G. Flügge, W. Haj Ahmad, O. Hlushchenko, T. Kress, T. Müller, A. Nowack, C. Pistone, O. Pooth, D. Roy, H. Sert, A. Stahl, M. Aldaya Martin, P. Asmuss, I. Babounikau, H. Bakhshiansohi, K. Beernaert, O. Behnke, A. Bermúdez Martínez, A. A. Bin Anuar, K. Borras, V. Botta, A. Campbell, A. Cardini, P. Connor, S. Consuegra Rodríguez, C. Contreras-Campana, V. Danilov, A. De Wit, M. M. Defranchis, C. Diez Pardos, D. Domínguez Damiani, G. Eckerlin, D. Eckstein, T. Eichhorn, A. Elwood, E. Eren, E. Gallo, A. Geiser, A. Grohsjean, M. Guthoff, M. Haranko, A. Harb, A. Jafari, N. Z. Jomhari, H. Jung, A. Kasem, M. Kasemann, H. Kaveh, J. Keaveney, C. Kleinwort, J. Knolle, D. Krücker, W. Lange, T. Lenz, J. Lidrych, K. Lipka, W. Lohmann, R. Mankel, I.-A. Melzer-Pellmann, A. B. Meyer, M. Meyer, M. Missiroli, J. Mnich, A. Mussgiller, V. Myronenko, D. Pérez Adán, S. K. Pflitsch, D. Pitzl, A. Raspereza, A. Saibel, M. Savitskyi, V. Scheurer, P. Schütze, C. Schwanenberger, R. Shevchenko, A. Singh, R. E. Sosa Ricardo, H. Tholen, O. Turkot, A. Vagnerini, M. Van De Klundert, R. Walsh, Y. Wen, K. Wichmann, C. Wissing, O. Zenaiev, R. Zlebcik, R. Aggleton, S. Bein, L. Benato, A. Benecke, T. Dreyer, A. Ebrahimi, F. Feindt, A. Fröhlich, C. Garbers, E. Garutti, D. Gonzalez, P. Gunnellini, J. Haller, A. Hinzmann, A. Karavdina, G. Kasieczka, R. Klanner, R. Kogler, N. Kovalchuk, S. Kurz, V. Kutzner, J. Lange, T. Lange, A. Malara, J. Multhaup, C. E. N. Niemeyer, A. Reimers, O. Rieger, P. Schleper, S. Schumann, J. Schwandt, J. Sonneveld, H. Stadie, G. Steinbrück, B. Vormwald, I. Zoi, M. Akbiyik, M. Baselga, S. Baur, T. Berger, E. Butz, R. Caspart, T. Chwalek, W. De Boer, A. Dierlamm, K. El Morabit, N. Faltermann, M. Giffels, A. Gottmann, M. A. Harrendorf, F. Hartmann, C. Heidecker, U. Husemann, S. Kudella, S. Maier, S. Mitra, M. U. Mozer, D. Müller, Th. Müller, M. Musich, A. Nürnberg, G. Quast, K. Rabbertz, D. Schäfer, M. Schröder, I. Shvetsov, H. J. Simonis, R. Ulrich, M. Wassmer, M. Weber, C. Wöhrmann, R. Wolf, S. Wozniewski, G. Anagnostou, P. Asenov, G. Daskalakis, T. Geralis, A. Kyriakis, D. Loukas, G. Paspalaki, M. Diamantopoulou, G. Karathanasis, P. Kontaxakis, A. Manousakis-katsikakis, A. Panagiotou, I. Papavergou, N. Saoulidou, A. Stakia, K. Theofilatos, K. Vellidis, E. Vourliotis, G. Bakas, K. Kousouris, I. Papakrivopoulos, G. Tsipolitis, I. Evangelou, C. Foudas, P. Gianneios, P. Katsoulis, P. Kokkas, S. Mallios, K. Manitara, N. Manthos, I. Papadopoulos, J. Strologas, F. A. Triantis, D. Tsitsonis, M. Bartók, R. Chudasama, M. Csanad, P. Major, K. Mandal, A. Mehta, G. Pasztor, O. Surányi, G. I. Veres, G. Bencze, C. Hajdu, D. Horvath, F. Sikler, V. Veszpremi, G. Vesztergombi, N. Beni, S. Czellar, J. Karancsi, J. Molnar, Z. Szillasi, P. Raics, D. Teyssier, Z. L. Trocsanyi, B. Ujvari, T. Csorgo, W. J. Metzger, F. Nemes, T. Novak, S. Choudhury, J. R. Komaragiri, P. C. Tiwari, S. Bahinipati, C. Kar, G. Kole, P. Mal, V. K. Muraleedharan Nair Bindhu, A. Nayak, D. K. Sahoo, S. K. Swain, S. Bansal, S. B. Beri, V. Bhatnagar, S. Chauhan, N. Dhingra, R. Gupta, A. Kaur, M. Kaur, S. Kaur, P. Kumari, M. Lohan, M. Meena, K. Sandeep, S. Sharma, J. B. Singh, A. K. Virdi, G. Walia, A. Bhardwaj, B. C. Choudhary, R. B. Garg, M. Gola, S. Keshri, A. Kumar, M. Naimuddin, P. Priyanka, K. Ranjan, A. Shah, R. Sharma, R. Bhardwaj, M. Bharti, R. Bhattacharya, S. Bhattacharya, U. Bhawandeep, D. Bhowmik, S. Dutta, S. Ghosh, B. Gomber, M. Maity, K. Mondal, S. Nandan, A. Purohit, P. K. Rout, G. Saha, S. Sarkar, T. Sarkar, M. Sharan, B. Singh, S. Thakur, P. K. Behera, P. Kalbhor, A. Muhammad, P. R. Pujahari, A. Sharma, A. K. Sikdar, D. Dutta, V. Jha, D. K. Mishra, P. K. Netrakanti, L. M. Pant, P. Shukla, T. Aziz, M. A. Bhat, S. Dugad, G. B. Mohanty, N. Sur, R. K. Verma, S. Banerjee, S. Bhattacharya, S. Chatterjee, P. Das, M. Guchait, S. Karmakar, S. Kumar, G. Majumder, K. Mazumdar, N. Sahoo, S. Sawant, S. Dube, B. Kansal, A. Kapoor, K. Kothekar, S. Pandey, A. Rane, A. Rastogi, S. Sharma, S. Chenarani, S. M. Etesami, M. Khakzad, M. Mohammadi Najafabadi, M. Naseri, F. Rezaei Hosseinabadi, M. Felcini, M. Grunewald, M. Abbrescia, R. Aly, C. Calabria, A. Colaleo, D. Creanza, L. Cristella, N. De Filippis, M. De Palma, A. Di Florio, W. Elmetenawee, L. Fiore, A. Gelmi, G. Iaselli, M. Ince, S. Lezki, G. Maggi, M. Maggi, J. A. Merlin, G. Miniello, S. My, S. Nuzzo, A. Pompili, G. Pugliese, R. Radogna, A. Ranieri, G. Selvaggi, L. Silvestris, F. M. Simone, R. Venditti, P. Verwilligen, G. Abbiendi, C. Battilana, D. Bonacorsi, L. Borgonovi, S. Braibant-Giacomelli, R. Campanini, P. Capiluppi, A. Castro, F. R. Cavallo, C. Ciocca, G. Codispoti, M. Cuffiani, G. M. Dallavalle, F. Fabbri, A. Fanfani, E. Fontanesi, P. Giacomelli, C. Grandi, L. Guiducci, F. Iemmi, S. Lo Meo, S. Marcellini, G. Masetti, F. L. Navarria, A. Perrotta, F. Primavera, A. M. Rossi, T. Rovelli, G. P. Siroli, N. Tosi, S. Albergo, S. Costa, A. Di Mattia, R. Potenza, A. Tricomi, C. Tuve, G. Barbagli, A. Cassese, R. Ceccarelli, V. Ciulli, C. Civinini, R. D’Alessandro, F. Fiori, E. Focardi, G. Latino, P. Lenzi, M. Meschini, S. Paoletti, G. Sguazzoni, L. Viliani, L. Benussi, S. Bianco, D. Piccolo, M. Bozzo, F. Ferro, R. Mulargia, E. Robutti, S. Tosi, A. Benaglia, A. Beschi, F. Brivio, V. Ciriolo, M. E. Dinardo, P. Dini, S. Gennai, A. Ghezzi, P. Govoni, L. Guzzi, M. Malberti, S. Malvezzi, D. Menasce, F. Monti, L. Moroni, M. Paganoni, D. Pedrini, S. Ragazzi, T. Tabarelli de Fatis, D. Valsecchi, D. Zuolo, S. Buontempo, N. Cavallo, A. De Iorio, A. Di Crescenzo, F. Fabozzi, F. Fienga, G. Galati, A. O. M. Iorio, L. Layer, L. Lista, S. Meola, P. Paolucci, B. Rossi, C. Sciacca, E. Voevodina, P. Azzi, N. Bacchetta, D. Bisello, A. Boletti, A. Bragagnolo, R. Carlin, P. Checchia, P. De Castro Manzano, T. Dorigo, U. Dosselli, F. Gasparini, U. Gasparini, A. Gozzelino, S. Y. Hoh, M. Margoni, A. T. Meneguzzo, J. Pazzini, M. Presilla, P. Ronchese, R. Rossin, F. Simonetto, A. Tiko, M. Tosi, M. Zanetti, P. Zotto, G. Zumerle, A. Braghieri, D. Fiorina, P. Montagna, S. P. Ratti, V. Re, M. Ressegotti, C. Riccardi, P. Salvini, I. Vai, P. Vitulo, M. Biasini, G. M. Bilei, D. Ciangottini, L. Fanò, P. Lariccia, R. Leonardi, E. Manoni, G. Mantovani, V. Mariani, M. Menichelli, A. Rossi, A. Santocchia, D. Spiga, K. Androsov, P. Azzurri, G. Bagliesi, V. Bertacchi, L. Bianchini, T. Boccali, R. Castaldi, M. A. Ciocci, R. Dell’Orso, S. Donato, L. Giannini, A. Giassi, M. T. Grippo, F. Ligabue, E. Manca, G. Mandorli, A. Messineo, F. Palla, A. Rizzi, G. Rolandi, S. Roy Chowdhury, A. Scribano, P. Spagnolo, R. Tenchini, G. Tonelli, N. Turini, A. Venturi, P. G. Verdini, F. Cavallari, M. Cipriani, D. Del Re, E. Di Marco, M. Diemoz, E. Longo, P. Meridiani, G. Organtini, F. Pandolfi, R. Paramatti, C. Quaranta, S. Rahatlou, C. Rovelli, F. Santanastasio, L. Soffi, N. Amapane, R. Arcidiacono, S. Argiro, M. Arneodo, N. Bartosik, R. Bellan, A. Bellora, C. Biino, A. Cappati, N. Cartiglia, S. Cometti, M. Costa, R. Covarelli, N. Demaria, B. Kiani, F. Legger, C. Mariotti, S. Maselli, E. Migliore, V. Monaco, E. Monteil, M. Monteno, M. M. Obertino, G. Ortona, L. Pacher, N. Pastrone, M. Pelliccioni, G. L. Pinna Angioni, A. Romero, M. Ruspa, R. Salvatico, V. Sola, A. Solano, D. Soldi, A. Staiano, D. Trocino, S. Belforte, V. Candelise, M. Casarsa, F. Cossutti, A. Da Rold, G. Della Ricca, F. Vazzoler, A. Zanetti, B. Kim, D. H. Kim, G. N. Kim, J. Lee, S. W. Lee, C. S. Moon, Y. D. Oh, S. I. Pak, S. Sekmen, D. C. Son, Y. C. Yang, H. Kim, D. H. Moon, G. Oh, B. Francois, T. J. Kim, J. Park, S. Cho, S. Choi, Y. Go, S. Ha, B. Hong, K. Lee, K. S. Lee, J. Lim, J. Park, S. K. Park, Y. Roh, J. Yoo, J. Goh, H. S. Kim, J. Almond, J. H. Bhyun, J. Choi, S. Jeon, J. Kim, J. S. Kim, H. Lee, K. Lee, S. Lee, K. Nam, M. Oh, S. B. Oh, B. C. Radburn-Smith, U. K. Yang, H. D. Yoo, I. Yoon, D. Jeon, J. H. Kim, J. S. H. Lee, I. C. Park, I. J. Watson, Y. Choi, C. Hwang, Y. Jeong, J. Lee, Y. Lee, I. Yu, V. Veckalns, V. Dudenas, A. Juodagalvis, A. Rinkevicius, G. Tamulaitis, J. Vaitkus, Z. A. Ibrahim, F. Mohamad Idris, W. A. T. Wan Abdullah, M. N. Yusli, Z. Zolkapli, J. F. Benitez, A. Castaneda Hernandez, J. A. Murillo Quijada, L. Valencia Palomo, H. Castilla-Valdez, E. De La Cruz-Burelo, I. Heredia-De La Cruz, R. Lopez-Fernandez, A. Sanchez-Hernandez, S. Carrillo Moreno, C. Oropeza Barrera, M. Ramirez-Garcia, F. Vazquez Valencia, J. Eysermans, I. Pedraza, H. A. Salazar Ibarguen, C. Uribe Estrada, A. Morelos Pineda, J. Mijuskovic, N. Raicevic, D. Krofcheck, S. Bheesette, P. H. Butler, P. Lujan, A. Ahmad, M. Ahmad, M. I. M. Awan, Q. Hassan, H. R. Hoorani, W. A. Khan, M. A. Shah, M. Shoaib, M. Waqas, V. Avati, L. Grzanka, M. Malawski, H. Bialkowska, M. Bluj, B. Boimska, M. Górski, M. Kazana, M. Szleper, P. Zalewski, K. Bunkowski, A. Byszuk, K. Doroba, A. Kalinowski, M. Konecki, J. Krolikowski, M. Olszewski, M. Walczak, M. Araujo, P. Bargassa, D. Bastos, A. Di Francesco, P. Faccioli, B. Galinhas, M. Gallinaro, J. Hollar, N. Leonardo, T. Niknejad, J. Seixas, K. Shchelina, G. Strong, O. Toldaiev, J. Varela, S. Afanasiev, P. Bunin, M. Gavrilenko, I. Golutvin, I. Gorbunov, A. Kamenev, V. Karjavine, A. Lanev, A. Malakhov, V. Matveev, P. Moisenz, V. Palichik, V. Perelygin, M. Savina, S. Shmatov, S. Shulha, N. Skatchkov, V. Smirnov, N. Voytishin, A. Zarubin, L. Chtchipounov, V. Golovtcov, Y. Ivanov, V. Kim, E. Kuznetsova, P. Levchenko, V. Murzin, V. Oreshkin, I. Smirnov, D. Sosnov, V. Sulimov, L. Uvarov, A. Vorobyev, Yu. Andreev, A. Dermenev, S. Gninenko, N. Golubev, A. Karneyeu, M. Kirsanov, N. Krasnikov, A. Pashenkov, D. Tlisov, A. Toropin, V. Epshteyn, V. Gavrilov, N. Lychkovskaya, A. Nikitenko, V. Popov, I. Pozdnyakov, G. Safronov, A. Spiridonov, A. Stepennov, M. Toms, E. Vlasov, A. Zhokin, T. Aushev, R. Chistov, M. Danilov, P. Parygin, S. Polikarpov, E. Tarkovskii, V. Andreev, M. Azarkin, I. Dremin, M. Kirakosyan, A. Terkulov, A. Belyaev, E. Boos, M. Dubinin, L. Dudko, A. Ershov, A. Gribushin, V. Klyukhin, O. Kodolova, I. Lokhtin, S. Obraztsov, S. Petrushanko, V. Savrin, A. Snigirev, A. Barnyakov, V. Blinov, T. Dimova, L. Kardapoltsev, Y. Skovpen, I. Azhgirey, I. Bayshev, S. Bitioukov, V. Kachanov, D. Konstantinov, P. Mandrik, V. Petrov, R. Ryutin, S. Slabospitskii, A. Sobol, S. Troshin, N. Tyurin, A. Uzunian, A. Volkov, A. Babaev, A. Iuzhakov, V. Okhotnikov, V. Borchsh, V. Ivanchenko, E. Tcherniaev, P. Adzic, P. Cirkovic, M. Dordevic, P. Milenovic, J. Milosevic, M. Stojanovic, M. Aguilar-Benitez, J. Alcaraz Maestre, A. Álvarez Fernández, I. Bachiller, M. Barrio Luna, C. F. Bedoya, J. A. Brochero Cifuentes, C. A. Carrillo Montoya, M. Cepeda, M. Cerrada, N. Colino, B. De La Cruz, A. Delgado Peris, J. P. Fernández Ramos, J. Flix, M. C. Fouz, O. Gonzalez Lopez, S. Goy Lopez, J. M. Hernandez, M. I. Josa, D. Moran, Á. Navarro Tobar, A. Pérez-Calero Yzquierdo, J. Puerta Pelayo, I. Redondo, L. Romero, S. Sánchez Navas, M. S. Soares, A. Triossi, C. Willmott, C. Albajar, J. F. de Trocóniz, R. Reyes-Almanza, B. Alvarez Gonzalez, J. Cuevas, C. Erice, J. Fernandez Menendez, S. Folgueras, I. Gonzalez Caballero, J. R. González Fernández, E. Palencia Cortezon, V. Rodríguez Bouza, S. Sanchez Cruz, I. J. Cabrillo, A. Calderon, B. Chazin Quero, J. Duarte Campderros, M. Fernandez, P. J. Fernández Manteca, A. García Alonso, G. Gomez, C. Martinez Rivero, P. Martinez Ruiz del Arbol, F. Matorras, J. Piedra Gomez, C. Prieels, T. Rodrigo, A. Ruiz-Jimeno, L. Russo, L. Scodellaro, I. Vila, J. M. Vizan Garcia, D. U. J. Sonnadara, W. G. D. Dharmaratna, N. Wickramage, D. Abbaneo, B. Akgun, E. Auffray, G. Auzinger, J. Baechler, P. Baillon, A. H. Ball, D. Barney, J. Bendavid, M. Bianco, A. Bocci, P. Bortignon, E. Bossini, E. Brondolin, T. Camporesi, A. Caratelli, G. Cerminara, E. Chapon, G. Cucciati, D. d’Enterria, A. Dabrowski, N. Daci, V. Daponte, A. David, O. Davignon, A. De Roeck, M. Deile, R. Di Maria, M. Dobson, M. Dünser, N. Dupont, A. Elliott-Peisert, N. Emriskova, F. Fallavollita, D. Fasanella, S. Fiorendi, G. Franzoni, J. Fulcher, W. Funk, S. Giani, D. Gigi, K. Gill, F. Glege, L. Gouskos, M. Gruchala, M. Guilbaud, D. Gulhan, J. Hegeman, C. Heidegger, Y. Iiyama, V. Innocente, T. James, P. Janot, O. Karacheban, J. Kaspar, J. Kieseler, M. Krammer, N. Kratochwil, C. Lange, P. Lecoq, C. Lourenço, L. Malgeri, M. Mannelli, A. Massironi, F. Meijers, S. Mersi, E. Meschi, F. Moortgat, M. Mulders, J. Ngadiuba, J. Niedziela, S. Nourbakhsh, S. Orfanelli, L. Orsini, F. Pantaleo, L. Pape, E. Perez, M. Peruzzi, A. Petrilli, G. Petrucciani, A. Pfeiffer, M. Pierini, F. M. Pitters, D. Rabady, A. Racz, M. Rieger, M. Rovere, H. Sakulin, J. Salfeld-Nebgen, S. Scarfi, C. Schäfer, C. Schwick, M. Selvaggi, A. Sharma, P. Silva, W. Snoeys, P. Sphicas, J. Steggemann, S. Summers, V. R. Tavolaro, D. Treille, A. Tsirou, G. P. Van Onsem, A. Vartak, M. Verzetti, W. D. Zeuner, L. Caminada, K. Deiters, W. Erdmann, R. Horisberger, Q. Ingram, H. C. Kaestli, D. Kotlinski, U. Langenegger, T. Rohe, M. Backhaus, P. Berger, N. Chernyavskaya, G. Dissertori, M. Dittmar, M. Donegà, C. Dorfer, T. A. Gómez Espinosa, C. Grab, D. Hits, W. Lustermann, R. A. Manzoni, M. T. Meinhard, F. Micheli, P. Musella, F. Nessi-Tedaldi, F. Pauss, G. Perrin, L. Perrozzi, S. Pigazzini, M. G. Ratti, M. Reichmann, C. Reissel, T. Reitenspiess, B. Ristic, D. Ruini, D. A. Sanz Becerra, M. Schönenberger, L. Shchutska, M. L. Vesterbacka Olsson, R. Wallny, D. H. Zhu, T. K. Aarrestad, C. Amsler, C. Botta, D. Brzhechko, M. F. Canelli, A. De Cosa, R. Del Burgo, B. Kilminster, S. Leontsinis, V. M. Mikuni, I. Neutelings, G. Rauco, P. Robmann, K. Schweiger, C. Seitz, Y. Takahashi, S. Wertz, A. Zucchetta, C. M. Kuo, W. Lin, A. Roy, S. S. Yu, P. Chang, Y. Chao, K. F. Chen, P. H. Chen, W.-S. Hou, Y. y. Li, R.-S. Lu, E. Paganis, A. Psallidas, A. Steen, B. Asavapibhop, C. Asawatangtrakuldee, N. Srimanobhas, N. Suwonjandee, A. Bat, F. Boran, A. Celik, S. Damarseckin, Z. S. Demiroglu, F. Dolek, C. Dozen, I. Dumanoglu, G. Gokbulut, E. G. Guler, Y. Guler, I. Hos, C. Isik, E. E. Kangal, O. Kara, A. Kayis Topaksu, U. Kiminsu, G. Onengut, K. Ozdemir, S. Ozturk, A. E. Simsek, U. G. Tok, S. Turkcapar, I. S. Zorbakir, C. Zorbilmez, B. Isildak, G. Karapinar, M. Yalvac, I. O. Atakisi, E. Gülmez, M. Kaya, O. Kaya, Ö. Özçelik, S. Tekten, E. A. Yetkin, A. Cakir, K. Cankocak, Y. Komurcu, S. Sen, S. Cerci, B. Kaynak, S. Ozkorucuklu, D. Sunar Cerci, B. Grynyov, L. Levchuk, E. Bhal, S. Bologna, J. J. Brooke, D. Burns, E. Clement, D. Cussans, H. Flacher, J. Goldstein, G. P. Heath, H. F. Heath, L. Kreczko, B. Krikler, S. Paramesvaran, T. Sakuma, S. Seif El Nasr-Storey, V. J. Smith, J. Taylor, A. Titterton, K. W. Bell, A. Belyaev, C. Brew, R. M. Brown, D. J. A. Cockerill, J. A. Coughlan, K. Harder, S. Harper, J. Linacre, K. Manolopoulos, D. M. Newbold, E. Olaiya, D. Petyt, T. Reis, T. Schuh, C. H. Shepherd-Themistocleous, A. Thea, I. R. Tomalin, T. Williams, R. Bainbridge, P. Bloch, J. Borg, S. Breeze, O. Buchmuller, A. Bundock, G. S. Chahal, D. Colling, P. Dauncey, G. Davies, M. Della Negra, P. Everaerts, G. Hall, G. Iles, M. Komm, L. Lyons, A.-M. Magnan, S. Malik, A. Martelli, V. Milosevic, A. Morton, J. Nash, V. Palladino, M. Pesaresi, D. M. Raymond, A. Richards, A. Rose, E. Scott, C. Seez, A. Shtipliyski, M. Stoye, T. Strebler, A. Tapper, K. Uchida, T. Virdee, N. Wardle, D. Winterbottom, A. G. Zecchinelli, S. C. Zenz, J. E. Cole, P. R. Hobson, A. Khan, P. Kyberd, C. K. Mackay, I. D. Reid, L. Teodorescu, S. Zahid, A. Brinkerhoff, K. Call, B. Caraway, J. Dittmann, K. Hatakeyama, C. Madrid, B. McMaster, N. Pastika, C. Smith, R. Bartek, A. Dominguez, R. Uniyal, A. M. Vargas Hernandez, A. Buccilli, S. I. Cooper, S. V. Gleyzer, C. Henderson, P. Rumerio, C. West, A. Albert, D. Arcaro, Z. Demiragli, D. Gastler, C. Richardson, J. Rohlf, D. Sperka, D. Spitzbart, I. Suarez, L. Sulak, D. Zou, G. Benelli, B. Burkle, X. Coubez, D. Cutts, Y. t. Duh, M. Hadley, U. Heintz, J. M. Hogan, K. H. M. Kwok, E. Laird, G. Landsberg, K. T. Lau, J. Lee, M. Narain, S. Sagir, R. Syarif, E. Usai, W. Y. Wong, D. Yu, W. Zhang, R. Band, C. Brainerd, R. Breedon, M. Calderon De La Barca Sanchez, M. Chertok, J. Conway, R. Conway, P. T. Cox, R. Erbacher, C. Flores, G. Funk, F. Jensen, W. Ko, O. Kukral, R. Lander, M. Mulhearn, D. Pellett, J. Pilot, M. Shi, D. Taylor, K. Tos, M. Tripathi, Z. Wang, F. Zhang, M. Bachtis, C. Bravo, R. Cousins, A. Dasgupta, A. Florent, J. Hauser, M. Ignatenko, N. Mccoll, W. A. Nash, S. Regnard, D. Saltzberg, C. Schnaible, B. Stone, V. Valuev, K. Burt, Y. Chen, R. Clare, J. W. Gary, S. M. A. Ghiasi Shirazi, G. Hanson, G. Karapostoli, O. R. Long, M. Olmedo Negrete, M. I. Paneva, W. Si, L. Wang, S. Wimpenny, B. R. Yates, Y. Zhang, J. G. Branson, P. Chang, S. Cittolin, S. Cooperstein, N. Deelen, M. Derdzinski, J. Duarte, R. Gerosa, D. Gilbert, B. Hashemi, D. Klein, V. Krutelyov, J. Letts, M. Masciovecchio, S. May, S. Padhi, M. Pieri, V. Sharma, M. Tadel, F. Würthwein, A. Yagil, G. Zevi Della Porta, N. Amin, R. Bhandari, C. Campagnari, M. Citron, V. Dutta, M. Franco Sevilla, J. Incandela, J. Ling, B. Marsh, H. Mei, A. Ovcharova, H. Qu, J. Richman, U. Sarica, D. Stuart, S. Wang, D. Anderson, A. Bornheim, O. Cerri, I. Dutta, J. M. Lawhorn, N. Lu, J. Mao, H. B. Newman, T. Q. Nguyen, J. Pata, M. Spiropulu, J. R. Vlimant, S. Xie, Z. Zhang, R. Y. Zhu, M. B. Andrews, T. Ferguson, T. Mudholkar, M. Paulini, M. Sun, I. Vorobiev, M. Weinberg, J. P. Cumalat, W. T. Ford, E. MacDonald, T. Mulholland, R. Patel, A. Perloff, K. Stenson, K. A. Ulmer, S. R. Wagner, J. Alexander, Y. Cheng, J. Chu, A. Datta, A. Frankenthal, K. Mcdermott, J. R. Patterson, D. Quach, A. Ryd, S. M. Tan, Z. Tao, J. Thom, P. Wittich, M. Zientek, S. Abdullin, M. Albrow, M. Alyari, G. Apollinari, A. Apresyan, A. Apyan, S. Banerjee, L. A. T. Bauerdick, A. Beretvas, D. Berry, J. Berryhill, P. C. Bhat, K. Burkett, J. N. Butler, A. Canepa, G. B. Cerati, H. W. K. Cheung, F. Chlebana, M. Cremonesi, V. D. Elvira, J. Freeman, Z. Gecse, E. Gottschalk, L. Gray, D. Green, S. Grünendahl, O. Gutsche, J. Hanlon, R. M. Harris, S. Hasegawa, R. Heller, J. Hirschauer, B. Jayatilaka, S. Jindariani, M. Johnson, U. Joshi, T. Klijnsma, B. Klima, M. J. Kortelainen, B. Kreis, S. Lammel, J. Lewis, D. Lincoln, R. Lipton, M. Liu, T. Liu, J. Lykken, K. Maeshima, J. M. Marraffino, D. Mason, P. McBride, P. Merkel, S. Mrenna, S. Nahn, V. O’Dell, V. Papadimitriou, K. Pedro, C. Pena, F. Ravera, A. Reinsvold Hall, L. Ristori, B. Schneider, E. Sexton-Kennedy, N. Smith, A. Soha, W. J. Spalding, L. Spiegel, S. Stoynev, J. Strait, L. Taylor, S. Tkaczyk, N. V. Tran, L. Uplegger, E. W. Vaandering, C. Vernieri, R. Vidal, M. Wang, H. A. Weber, A. Woodard, D. Acosta, P. Avery, D. Bourilkov, L. Cadamuro, V. Cherepanov, F. Errico, R. D. Field, D. Guerrero, B. M. Joshi, M. Kim, J. Konigsberg, A. Korytov, K. H. Lo, K. Matchev, N. Menendez, G. Mitselmakher, D. Rosenzweig, K. Shi, J. Wang, S. Wang, X. Zuo, Y. R. Joshi, T. Adams, A. Askew, S. Hagopian, V. Hagopian, K. F. Johnson, R. Khurana, T. Kolberg, G. Martinez, T. Perry, H. Prosper, C. Schiber, R. Yohay, J. Zhang, M. M. Baarmand, M. Hohlmann, D. Noonan, M. Rahmani, M. Saunders, F. Yumiceva, M. R. Adams, L. Apanasevich, R. R. Betts, R. Cavanaugh, X. Chen, S. Dittmer, O. Evdokimov, C. E. Gerber, D. A. Hangal, D. J. Hofman, V. Kumar, C. Mills, T. Roy, M. B. Tonjes, N. Varelas, J. Viinikainen, H. Wang, X. Wang, Z. Wu, M. Alhusseini, B. Bilki, K. Dilsiz, S. Durgut, R. P. Gandrajula, M. Haytmyradov, V. Khristenko, O. K. Köseyan, J.-P. Merlo, A. Mestvirishvili, A. Moeller, J. Nachtman, H. Ogul, Y. Onel, F. Ozok, A. Penzo, C. Snyder, E. Tiras, J. Wetzel, B. Blumenfeld, A. Cocoros, N. Eminizer, A. V. Gritsan, W. T. Hung, S. Kyriacou, P. Maksimovic, J. Roskes, M. Swartz, T.Á. Vámi, C. Baldenegro Barrera, P. Baringer, A. Bean, S. Boren, A. Bylinkin, T. Isidori, S. Khalil, J. King, G. Krintiras, A. Kropivnitskaya, C. Lindsey, D. Majumder, W. Mcbrayer, N. Minafra, M. Murray, C. Rogan, C. Royon, S. Sanders, E. Schmitz, J. D. Tapia Takaki, Q. Wang, J. Williams, G. Wilson, S. Duric, A. Ivanov, K. Kaadze, D. Kim, Y. Maravin, D. R. Mendis, T. Mitchell, A. Modak, A. Mohammadi, F. Rebassoo, D. Wright, A. Baden, O. Baron, A. Belloni, S. C. Eno, Y. Feng, N. J. Hadley, S. Jabeen, G. Y. Jeng, R. G. Kellogg, A. C. Mignerey, S. Nabili, F. Ricci-Tam, M. Seidel, Y. H. Shin, A. Skuja, S. C. Tonwar, K. Wong, D. Abercrombie, B. Allen, R. Bi, S. Brandt, W. Busza, I. A. Cali, M. D’Alfonso, G. Gomez Ceballos, M. Goncharov, P. Harris, D. Hsu, M. Hu, M. Klute, D. Kovalskyi, Y.-J. Lee, P. D. Luckey, B. Maier, A. C. Marini, C. Mcginn, C. Mironov, S. Narayanan, X. Niu, C. Paus, D. Rankin, C. Roland, G. Roland, Z. Shi, G. S. F. Stephans, K. Sumorok, K. Tatar, D. Velicanu, J. Wang, T. W. Wang, B. Wyslouch, R. M. Chatterjee, A. Evans, S. Guts, P. Hansen, J. Hiltbrand, Sh. Jain, Y. Kubota, Z. Lesko, J. Mans, M. Revering, R. Rusack, R. Saradhy, N. Schroeder, N. Strobbe, M. A. Wadud, J. G. Acosta, S. Oliveros, K. Bloom, S. Chauhan, D. R. Claes, C. Fangmeier, L. Finco, F. Golf, R. Kamalieddin, I. Kravchenko, J. E. Siado, G. R. Snow, B. Stieger, W. Tabb, G. Agarwal, C. Harrington, I. Iashvili, A. Kharchilava, C. McLean, D. Nguyen, A. Parker, J. Pekkanen, S. Rappoccio, B. Roozbahani, G. Alverson, E. Barberis, C. Freer, Y. Haddad, A. Hortiangtham, G. Madigan, B. Marzocchi, D. M. Morse, T. Orimoto, L. Skinnari, A. Tishelman-Charny, T. Wamorkar, B. Wang, A. Wisecarver, D. Wood, S. Bhattacharya, J. Bueghly, G. Fedi, A. Gilbert, T. Gunter, K. A. Hahn, N. Odell, M. H. Schmitt, K. Sung, M. Velasco, R. Bucci, N. Dev, R. Goldouzian, M. Hildreth, K. Hurtado Anampa, C. Jessop, D. J. Karmgard, K. Lannon, W. Li, N. Loukas, N. Marinelli, I. Mcalister, F. Meng, Y. Musienko, R. Ruchti, P. Siddireddy, G. Smith, S. Taroni, M. Wayne, A. Wightman, M. Wolf, J. Alimena, B. Bylsma, L. S. Durkin, B. Francis, C. Hill, W. Ji, A. Lefeld, T. Y. Ling, B. L. Winer, G. Dezoort, P. Elmer, J. Hardenbrook, N. Haubrich, S. Higginbotham, A. Kalogeropoulos, S. Kwan, D. Lange, M. T. Lucchini, J. Luo, D. Marlow, K. Mei, I. Ojalvo, J. Olsen, C. Palmer, P. Piroué, D. Stickland, C. Tully, S. Malik, S. Norberg, A. Barker, V. E. Barnes, R. Chawla, S. Das, L. Gutay, M. Jones, A. W. Jung, B. Mahakud, D. H. Miller, G. Negro, N. Neumeister, C. C. Peng, S. Piperov, H. Qiu, J. F. Schulte, N. Trevisani, F. Wang, R. Xiao, W. Xie, T. Cheng, J. Dolen, N. Parashar, A. Baty, U. Behrens, S. Dildick, K. M. Ecklund, S. Freed, F. J. M. Geurts, M. Kilpatrick, A. Kumar, W. Li, B. P. Padley, R. Redjimi, J. Roberts, J. Rorie, W. Shi, A. G. Stahl Leiton, Z. Tu, A. Zhang, A. Bodek, P. de Barbaro, R. Demina, J. L. Dulemba, C. Fallon, T. Ferbel, M. Galanti, A. Garcia-Bellido, O. Hindrichs, A. Khukhunaishvili, E. Ranken, R. Taus, B. Chiarito, J. P. Chou, A. Gandrakota, Y. Gershtein, E. Halkiadakis, A. Hart, M. Heindl, E. Hughes, S. Kaplan, I. Laflotte, A. Lath, R. Montalvo, K. Nash, M. Osherson, S. Salur, S. Schnetzer, S. Somalwar, R. Stone, S. Thomas, H. Acharya, A. G. Delannoy, S. Spanier, O. Bouhali, M. Dalchenko, M. De Mattia, A. Delgado, R. Eusebi, J. Gilmore, T. Huang, T. Kamon, H. Kim, S. Luo, S. Malhotra, D. Marley, R. Mueller, D. Overton, L. Perniè, D. Rathjens, A. Safonov, N. Akchurin, J. Damgov, F. De Guio, V. Hegde, S. Kunori, K. Lamichhane, S. W. Lee, T. Mengke, S. Muthumuni, T. Peltola, S. Undleeb, I. Volobouev, Z. Wang, A. Whitbeck, S. Greene, A. Gurrola, R. Janjam, W. Johns, C. Maguire, A. Melo, H. Ni, K. Padeken, F. Romeo, P. Sheldon, S. Tuo, J. Velkovska, M. Verweij, M. W. Arenton, P. Barria, B. Cox, G. Cummings, J. Hakala, R. Hirosky, M. Joyce, A. Ledovskoy, C. Neu, B. Tannenwald, Y. Wang, E. Wolfe, F. Xia, R. Harr, P. E. Karchin, N. Poudyal, J. Sturdy, P. Thapa, K. Black, T. Bose, J. Buchanan, C. Caillol, D. Carlsmith, S. Dasu, I. De Bruyn, L. Dodd, C. Galloni, H. He, M. Herndon, A. Hervé, U. Hussain, A. Lanaro, A. Loeliger, K. Long, R. Loveless, J. Madhusudanan Sreekala, A. Mallampalli, D. Pinna, T. Ruggles, A. Savin, V. Sharma, W. H. Smith, D. Teague, S. Trembath-reichert, CMS Collaboration

**Affiliations:** 1 Yerevan Physics Institute, Yerevan, Armenia; 2 Institut für Hochenergiephysik, Wien, Austria; 3 Institute for Nuclear Problems, Minsk, Belarus; 4 Universiteit Antwerpen, Antwerpen, Belgium; 5 Vrije Universiteit Brussel, Brussels, Belgium; 6 Université Libre de Bruxelles, Brussels, Belgium; 7 Ghent University, Ghent, Belgium; 8 Université Catholique de Louvain, Louvain-la-Neuve, Belgium; 9 Centro Brasileiro de Pesquisas Fisicas, Rio de Janeiro, Brazil; 10 Universidade do Estado do Rio de Janeiro, Rio de Janeiro, Brazil; 11 Universidade Estadual Paulista, Universidade Federal do ABC, São Paulo, Brazil; 12 Institute for Nuclear Research and Nuclear Energy, Bulgarian Academy of Sciences, Sofia, Bulgaria; 13 University of Sofia, Sofia, Bulgaria; 14 Beihang University, Beijing, China; 15 Department of Physics, Tsinghua University, Beijing, China; 16 Institute of High Energy Physics, Beijing, China; 17 State Key Laboratory of Nuclear Physics and Technology, Peking University, Beijing, China; 18 Zhejiang University, Hangzhou, China; 19 Universidad de Los Andes, Bogotá, Colombia; 20 Universidad de Antioquia, Medellin, Colombia; 21 University of Split, Faculty of Electrical Engineering, Mechanical Engineering and Naval Architecture, Split, Croatia; 22 University of Split, Faculty of Science, Split, Croatia; 23 Institute Rudjer Boskovic, Zagreb, Croatia; 24 University of Cyprus, Nicosia, Cyprus; 25 Charles University, Prague, Czech Republic; 26 Escuela Politecnica Nacional, Quito, Ecuador; 27 Universidad San Francisco de Quito, Quito, Ecuador; 28 Academy of Scientific Research and Technology of the Arab Republic of Egypt, Egyptian Network of High Energy Physics, Cairo, Egypt; 29 National Institute of Chemical Physics and Biophysics, Tallinn, Estonia; 30 Department of Physics, University of Helsinki, Helsinki, Finland; 31 Helsinki Institute of Physics, Helsinki, Finland; 32 Lappeenranta University of Technology, Lappeenranta, Finland; 33 IRFU, CEA, Université Paris-Saclay, Gif-sur-Yvette, France; 34 Laboratoire Leprince-Ringuet, CNRS/IN2P3, Ecole Polytechnique, Institut Polytechnique de Paris, Paris, France; 35 Université de Strasbourg, CNRS, IPHC UMR 7178, Strasbourg, France; 36 Centre de Calcul de l’Institut National de Physique Nucleaire et de Physique des Particules, CNRS/IN2P3, Villeurbanne, France; 37 CNRS-IN2P3, Institut de Physique Nucléaire de Lyon, Université de Lyon, Université Claude Bernard Lyon 1, Villeurbanne, France; 38 Georgian Technical University, Tbilisi, Georgia; 39 Tbilisi State University, Tbilisi, Georgia; 40 RWTH Aachen University, I. Physikalisches Institut, Aachen, Germany; 41 RWTH Aachen University, III. Physikalisches Institut A, Aachen, Germany; 42 RWTH Aachen University, III. Physikalisches Institut B, Aachen, Germany; 43 Deutsches Elektronen-Synchrotron, Hamburg, Germany; 44 University of Hamburg, Hamburg, Germany; 45 Karlsruher Institut fuer Technologie, Karlsruhe, Germany; 46 Institute of Nuclear and Particle Physics (INPP), NCSR Demokritos, Aghia Paraskevi, Greece; 47 National and Kapodistrian University of Athens, Athens, Greece; 48 National Technical University of Athens, Athens, Greece; 49 University of Ioánnina, Ioánnina, Greece; 50 MTA-ELTE Lendület CMS Particle and Nuclear Physics Group, Eötvös Loránd University, Budapest, Hungary; 51 Wigner Research Centre for Physics, Budapest, Hungary; 52 Institute of Nuclear Research ATOMKI, Debrecen, Hungary; 53 Institute of Physics, University of Debrecen, Debrecen, Hungary; 54 Eszterhazy Karoly University, Karoly Robert Campus, Gyongyos, Hungary; 55 Indian Institute of Science (IISc), Bangalore, India; 56 National Institute of Science Education and Research, HBNI, Bhubaneswar, India; 57 Panjab University, Chandigarh, India; 58 University of Delhi, Delhi, India; 59 Saha Institute of Nuclear Physics, HBNI, Kolkata, India; 60 Indian Institute of Technology Madras, Chennai, India; 61 Bhabha Atomic Research Centre, Mumbai, India; 62 Tata Institute of Fundamental Research-A, Mumbai, India; 63 Tata Institute of Fundamental Research-B, Mumbai, India; 64 Indian Institute of Science Education and Research (IISER), Pune, India; 65 Institute for Research in Fundamental Sciences (IPM), Tehran, Iran; 66 University College Dublin, Dublin, Ireland; 67 INFN Sezione di Bari, Università di Bari, Politecnico di Bari, Bari, Italy; 68 INFN Sezione di Bologna, Università di Bologna, Bologna, Italy; 69 INFN Sezione di Catania, Università di Catania, Catania, Italy; 70 INFN Sezione di Firenze, Università di Firenze, Firenze, Italy; 71 INFN Laboratori Nazionali di Frascati, Frascati, Italy; 72 INFN Sezione di Genova, Università di Genova, Genoa, Italy; 73 INFN Sezione di Milano-Bicocca, Università di Milano-Bicocca, Milan, Italy; 74 INFN Sezione di Napoli, Università di Napoli ‘Federico II’, Napoli, Italy, Università della Basilicata, Potenza, Italy, Università G. Marconi, Rome, Italy; 75 INFN Sezione di Padova, Università di Padova, Padova, Italy, Università di Trento, Trento, Italy; 76 INFN Sezione di Pavia, Università di Pavia, Pavia, Italy; 77 INFN Sezione di Perugia, Università di Perugia, Perugia, Italy; 78 INFN Sezione di Pisa, Università di Pisa, Scuola Normale Superiore di Pisa, Pisa, Italy; 79 INFN Sezione di Roma, Sapienza Università di Roma, Rome, Italy; 80 INFN Sezione di Torino, Università di Torino, Torino, Italy, Università del Piemonte Orientale, Novara, Italy; 81 INFN Sezione di Trieste, Università di Trieste, Trieste, Italy; 82 Kyungpook National University, Daegu, Korea; 83 Chonnam National University, Institute for Universe and Elementary Particles, Kwangju, Korea; 84 Hanyang University, Seoul, Korea; 85 Korea University, Seoul, Korea; 86 Department of Physics, Kyung Hee University, Seoul, Korea; 87 Sejong University, Seoul, Korea; 88 Seoul National University, Seoul, Korea; 89 University of Seoul, Seoul, Korea; 90 Sungkyunkwan University, Suwon, Korea; 91 Riga Technical University, Riga, Latvia; 92 Vilnius University, Vilnius, Lithuania; 93 National Centre for Particle Physics, Universiti Malaya, Kuala Lumpur, Malaysia; 94 Universidad de Sonora (UNISON), Hermosillo, Mexico; 95 Centro de Investigacion y de Estudios Avanzados del IPN, Mexico City, Mexico; 96 Universidad Iberoamericana, Mexico City, Mexico; 97 Benemerita Universidad Autonoma de Puebla, Puebla, Mexico; 98 Universidad Autónoma de San Luis Potosí, San Luis Potosí, Mexico; 99 University of Montenegro, Podgorica, Montenegro; 100 University of Auckland, Auckland, New Zealand; 101 University of Canterbury, Christchurch, New Zealand; 102 National Centre for Physics, Quaid-I-Azam University, Islamabad, Pakistan; 103 AGH University of Science and Technology Faculty of Computer Science, Electronics and Telecommunications, Kraków, Poland; 104 National Centre for Nuclear Research, Swierk, Poland; 105 Institute of Experimental Physics, Faculty of Physics, University of Warsaw, Warsaw, Poland; 106 Laboratório de Instrumentação e Física Experimental de Partículas, Lisbon, Portugal; 107 Joint Institute for Nuclear Research, Dubna, Russia; 108 Petersburg Nuclear Physics Institute, Gatchina (St. Petersburg), Russia; 109 Institute for Nuclear Research, Moscow, Russia; 110 Institute for Theoretical and Experimental Physics named by A.I. Alikhanov of NRC ‘Kurchatov Institute’, Moscow, Russia; 111 Moscow Institute of Physics and Technology, Moscow, Russia; 112 National Research Nuclear University ‘Moscow Engineering Physics Institute’ (MEPhI), Moscow, Russia; 113 P.N. Lebedev Physical Institute, Moscow, Russia; 114 Skobeltsyn Institute of Nuclear Physics, Lomonosov Moscow State University, Moscow, Russia; 115 Novosibirsk State University (NSU), Novosibirsk, Russia; 116 Institute for High Energy Physics of National Research Centre ‘Kurchatov Institute’, Protvino, Russia; 117 National Research Tomsk Polytechnic University, Tomsk, Russia; 118 Tomsk State University, Tomsk, Russia; 119 Faculty of Physics and VINCA Institute of Nuclear Sciences, University of Belgrade, Belgrade, Serbia; 120 Centro de Investigaciones Energéticas Medioambientales y Tecnológicas (CIEMAT), Madrid, Spain; 121 Universidad Autónoma de Madrid, Madrid, Spain; 122 Instituto Universitario de Ciencias y Tecnologías Espaciales de Asturias (ICTEA), Universidad de Oviedo, Oviedo, Spain; 123 Instituto de Física de Cantabria (IFCA), CSIC-Universidad de Cantabria, Santander, Spain; 124 University of Colombo, Colombo, Sri Lanka; 125 Department of Physics, University of Ruhuna, Matara, Sri Lanka; 126 CERN, European Organization for Nuclear Research, Geneva, Switzerland; 127 Paul Scherrer Institut, Villigen, Switzerland; 128 ETH Zurich-Institute for Particle Physics and Astrophysics (IPA), Zurich, Switzerland; 129 Universität Zürich, Zurich, Switzerland; 130 National Central University, Chung-Li, Taiwan; 131 National Taiwan University (NTU), Taipei, Taiwan; 132 Department of Physics, Faculty of Science, Chulalongkorn University, Bangkok, Thailand; 133 Physics Department, Science and Art Faculty, Çukurova University, Adana, Turkey; 134 Physics Department, Middle East Technical University, Ankara, Turkey; 135 Bogazici University, Istanbul, Turkey; 136 Istanbul Technical University, Istanbul, Turkey; 137 Istanbul University, Istanbul, Turkey; 138 Institute for Scintillation Materials of National Academy of Science of Ukraine, Kharkov, Ukraine; 139 National Scientific Center, Kharkov Institute of Physics and Technology, Kharkov, Ukraine; 140 University of Bristol, Bristol, UK; 141 Rutherford Appleton Laboratory, Didcot, UK; 142 Imperial College, London, UK; 143 Brunel University, Uxbridge, UK; 144 Baylor University, Waco, USA; 145 Catholic University of America, Washington, DC USA; 146 The University of Alabama, Tuscaloosa, USA; 147 Boston University, Boston, USA; 148 Brown University, Providence, USA; 149 University of California, Davis, Davis, USA; 150 University of California, Los Angeles, USA; 151 University of California, Riverside, Riverside, USA; 152 University of California, San Diego, La Jolla, USA; 153 Department of Physics, University of California, Santa Barbara, Santa Barbara, USA; 154 California Institute of Technology, Pasadena, USA; 155 Carnegie Mellon University, Pittsburgh, USA; 156 University of Colorado Boulder, Boulder, USA; 157 Cornell University, Ithaca, USA; 158 Fermi National Accelerator Laboratory, Batavia, USA; 159 University of Florida, Gainesville, USA; 160 Florida International University, Miami, USA; 161 Florida State University, Tallahassee, USA; 162 Florida Institute of Technology, Melbourne, USA; 163 University of Illinois at Chicago (UIC), Chicago, USA; 164 The University of Iowa, Iowa City, USA; 165 Johns Hopkins University, Baltimore, USA; 166 The University of Kansas, Lawrence, USA; 167 Kansas State University, Manhattan, USA; 168 Lawrence Livermore National Laboratory, Livermore, USA; 169 University of Maryland, College Park, USA; 170 Massachusetts Institute of Technology, Cambridge, USA; 171 University of Minnesota, Minneapolis, USA; 172 University of Mississippi, Oxford, USA; 173 University of Nebraska-Lincoln, Lincoln, USA; 174 State University of New York at Buffalo, Buffalo, USA; 175 Northeastern University, Boston, USA; 176 Northwestern University, Evanston, USA; 177 University of Notre Dame, Notre Dame, USA; 178 The Ohio State University, Columbus, USA; 179 Princeton University, Princeton, USA; 180 University of Puerto Rico, Mayaguez, USA; 181 Purdue University, West Lafayette, USA; 182 Purdue University Northwest, Hammond, USA; 183 Rice University, Houston, USA; 184 University of Rochester, Rochester, USA; 185 Rutgers, The State University of New Jersey, Piscataway, USA; 186 University of Tennessee, Knoxville, USA; 187 Texas A&M University, College Station, USA; 188 Texas Tech University, Lubbock, USA; 189 Vanderbilt University, Nashville, USA; 190 University of Virginia, Charlottesville, USA; 191 Wayne State University, Detroit, USA; 192 University of Wisconsin-Madison, Madison, WI USA; 193 CERN, 1211 Geneva 23, Switzerland

## Abstract

A data sample of events from proton-proton collisions with at least two jets, and two isolated same-sign or three or more charged leptons, is studied in a search for signatures of new physics phenomena. The data correspond to an integrated luminosity of $137{\,{\text {fb}}^{-1}}$ at a center-of-mass energy of $13\,{\text {TeV}}$, collected in 2016–2018 by the CMS experiment at the LHC. The search is performed using a total of 168 signal regions defined using several kinematic variables. The properties of the events are found to be consistent with the expectations from standard model processes. Exclusion limits at 95% confidence level are set on cross sections for the pair production of gluinos or squarks for various decay scenarios in the context of supersymmetric models conserving or violating R parity. The observed lower mass limits are as large as $2.1\,{\text {TeV}}$ for gluinos and $0.9\,{\text {TeV}}$ for top and bottom squarks. To facilitate reinterpretations, model-independent limits are provided in a set of simplified signal regions.

## Introduction

In the standard model (SM), the production of multiple jets in conjunction with two same-sign (SS) or three or more charged leptons is a very rare process in proton-proton (${\text {p}}{\text {p}}$) collisions. These final states provide a promising starting point in the search for physics beyond the SM (BSM). Many models attempting to address the shortcomings of the SM lead to such signatures. Examples include the production of supersymmetric (SUSY) particles [1, 2], SS top quark pairs [3, 4], scalar gluons (sgluons) [5, 6], heavy scalar bosons of extended Higgs sectors [7, 8], Majorana neutrinos [9], and vector-like quarks [10].

In SUSY models [11–19], the decay chain of pair-produced gluinos or squarks can contain multiple ${\text {W}}$ or ${\text {Z}}$ bosons, with the potential to have at least one pair of SS ${\text {W}}$ bosons. Such a decay chain is realized, for example in gluino pair production, when a gluino decays into a top quark-antiquark pair and a neutralino, or into a pair of quarks and a chargino that subsequently decays into a ${\text {W}}$ boson and a neutralino. In R parity [20] conserving (RPC) scenarios, the lightest SUSY particle is neutral and stable and escapes detection, leading to an imbalance in the measured transverse momentum. The magnitude of the missing transverse momentum strongly depends on the details of the model, and in particular on the mass spectrum of the particles involved. Scenarios with R parity violation (RPV) [21, 22] additionally allow decays of SUSY particles into SM particles only, leading in many cases to signatures with little or no missing transverse momentum. For many SUSY models, the SS and multilepton signatures provide complementarity with searches in the zero- or one-lepton final states, and they are particularly suitable for probing compressed mass spectra and other scenarios involving low-momentum leptons or low missing transverse momentum. Both the ATLAS [23] and CMS [24, 25] Collaborations have carried out searches in these channels using LHC data collected up to and including 2016. The ATLAS Collaboration has also recently released a search with the full data set recorded between 2015 and 2018 [26].

In this paper, we extend and refine the searches described in Refs. [24, 25] using a larger data set of ${\text {p}}{\text {p}}$ collisions at $\sqrt{s} = 13\,{\text {TeV}}$ recorded by the CMS detector at the CERN LHC in 2016–2018, corresponding to an integrated luminosity of $137{\,{\text {fb}}^{-1}}$. We base our search on an initial selection of events with at least two hadronic jets and two SS or three or more light leptons (electrons and muons), including those from leptonic decays of $\tau$ leptons. Several signal regions (SRs) are then constructed with requirements on variables such as the number of leptons, the number of jets (possibly identified as originating from ${\text {b}}$ quarks), and the magnitude of missing transverse momentum. A simultaneous comparison of the observed and SM plus BSM expected event yields in all SRs is performed to constrain the BSM models described in Sect. 2. After a brief description of the CMS experiment in Sect. 3, we present the details of the search strategy and event selection in Sect. 4 and discuss the various relevant backgrounds from SM processes in Sect. 5. The systematic uncertainties considered in the analysis are presented in Sect. 6. In Sect. 7, the observed yields are compared to the background expectation and the results are interpreted to constrain the various BSM models introduced earlier. Model independent limits are also derived. Finally, the main results are summarized in Sect. 8.

## Background and signal simulation

Monte Carlo (MC) simulations are used to study the SM backgrounds and to estimate the event selection efficiency of the BSM signals under consideration. Three sets of simulated events for each process are used in order to match the different data taking conditions in 2016, 2017, and 2018.

The hard scattering process of the dominant backgrounds estimated from simulation (including the $\hbox {t}{\bar{\hbox {t}}} {\text {W}}$, $\hbox {t}{\bar{\hbox {t}}} {\text {Z}}$ and ${\text {W}}{\text {Z}}$ contributions) is simulated with the MadGraph 5_amc@nlo 2.2.2 (2.4.2) [27–29] generator for 2016 (2017 and 2018) conditions. An exception is the ${\text {W}}{\text {Z}}$ process for the 2016 conditions that, as with a few subdominant backgrounds, is simulated using the powheg  v2 [30–34] next-to-leading order (NLO) generator. Samples of signal events, as well as of SS ${\text {W}}$ boson pairs and other very rare SM processes, are generated at leading order (LO) accuracy with MadGraph 5_amc@nlo, with up to two additional partons in the matrix element calculations. The set of parton distribution functions (PDFs) used was NNPDF3.0 [35] for the 2016 simulation and NNPDF3.1 [36] for the 2017 and 2018 simulations.

Parton showering and hadronization, as well as the double parton scattering production of ${\text {W}}^{\pm } {\text {W}}^{\pm }$, are described using the pythia  8.230 generator [37] with the CUETP8M1 (CP5) underlying event tune for 2016 (2017 and 2018) simulation [38–40]. The response of the CMS detector is modeled using the Geant4 program [41] for SM background samples, while the CMS fast simulation package [42, 43] is used for signal samples.

To improve the MadGraph 5_amc@nlo modeling of the multiplicity of additional jets from initial-state radiation (ISR), 2016 MC events are reweighted according to the number of ISR jets ($N_J^\text {ISR}$). The reweighting factors are extracted from a study of the light-flavor jet multiplicity in dilepton $\hbox {t}{\bar{\hbox {t}}}$ events. They vary between 0.92 and 0.77 for $N_J^\text {ISR}$ between 1 and 4, with one half of the deviation from unity taken as the systematic uncertainty. This reweighting is not necessary for the 2017 and 2018 MC samples that are generated using an updated pythia tune.

**Fig. 1:**
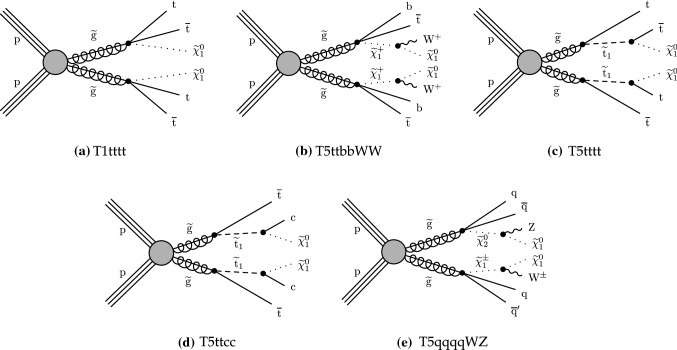
Diagrams illustrating the simplified RPC SUSY models with gluino production considered in this analysis

**Fig. 2:**
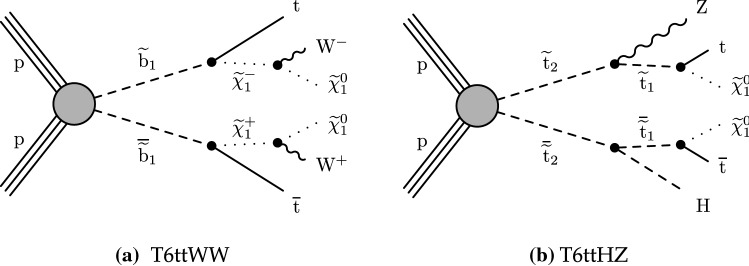
Diagrams illustrating the simplified RPC SUSY models with squark production considered in this analysis

**Fig. 3:**
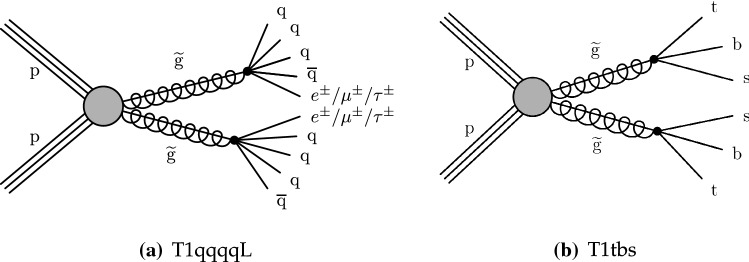
Diagrams illustrating the two simplified RPV SUSY models considered in this analysis

The phenomenology of a given SUSY model strongly depends on its underlying details such as the masses of the SUSY particles and their couplings with the SM particles and each other, many of which can be free parameters. The signal models used by this search are simplified SUSY models [44, 45] of either gluino or squark pair production, followed by a variety of RPC (Figs. 1, 2) or RPV (Fig. 3) decays and where several leptons can arise in the final state. Production cross sections are calculated at approximate next-to-next-to-leading order plus next-to-next-to-leading logarithmic (NNLO+NNLL) accuracy [46–58]. The branching fractions for the decays shown are assumed to be 100%, unless otherwise specified, and all decays are assumed to be prompt.

Gluino pair production models giving rise to signatures with up to four ${\text {b}}$ quarks and up to four ${\text {W}}$ bosons are shown in Fig. 1. In these models, the gluino decays to the lightest squark (${\tilde{\text {g}}} \rightarrow {\tilde{\text {q}}} {\text {q}}$), which in turn decays to same-flavor (${\tilde{\text {q}}} \rightarrow {\text {q}}{\tilde{\chi }}^{0}_{1}$) or different-flavor (${\tilde{\text {q}}} \rightarrow {\text {q}}' {\tilde{\chi }}^\pm _{1}$) quarks. The chargino (${\tilde{\chi }}^\pm _{1}$) decays to a ${\text {W}}$ boson and a neutralino (${\tilde{\chi }}^{0}_{1}$) via ${\tilde{\chi }}^\pm _{1} \rightarrow {\text {W}}^{\pm } {\tilde{\chi }}^{0}_{1}$, where the ${\tilde{\chi }}^{0}_{1}$ is taken to be the lightest stable SUSY particle and escapes detection.

The first scenario, denoted by $\mathrm {T}1{{\text {t t t t}}}$ and displayed in Fig. 1a, includes an off-shell top squark (${\tilde{\text {t}}}$) leading to the three-body decay of the gluino, ${\tilde{\text {g}}} \rightarrow \hbox {t}{\bar{\hbox {t}}} {\tilde{\chi }}^{0}_{1}$, resulting in events with four ${\text {W}}$ bosons and four ${\text {b}}$ quarks. Figure 1b presents a similar model ($\mathrm {T}5{{\text {t t b b W W}}}$) where the gluino decay results in a chargino that further decays into a neutralino and a ${\text {W}}$ boson. The model shown in Fig. 1c ($\mathrm {T}5{{\text {t t t t}}}$) is the same as $\mathrm {T}1{{\text {t t t t}}}$ except that the intermediate top squark is on-shell. The mass splitting between the ${\tilde{\text {t}}}$ and the ${\tilde{\chi }}^{0}_{1}$ is taken to be $m_{{\tilde{\text {t}}}} - m_{{\tilde{\chi }}^{0}_{1}} = m_{{\text {t}}}$, where $m_{{\text {t}}}$ is the top quark mass. This choice maximizes the kinematic differences between this model and $\mathrm {T}1{{\text {t t t t}}}$, and also corresponds to one of the most challenging regions of parameter space for the observation of the ${\tilde{\text {t}}} \rightarrow {\text {t}}{\tilde{\chi }}^{0}_{1}$ decay since the neutralino is produced at rest in the top squark rest frame. The decay chain of Fig. 1d ($\mathrm {T}5{{\text {t t c c}}}$) is identical to that of $\mathrm {T}5{{\text {t t t t}}}$ except that the ${\tilde{\text {t}}}$ decay involves a ${\text {c}}$quark. In Fig. 1e, the decay process includes a virtual light-flavor squark, leading to three-body decays of ${\tilde{\text {g}}} \rightarrow {\text {q q}}' {\tilde{\chi }}^\pm _{1}$ or ${\tilde{\text {g}}} \rightarrow {\text {q q}}' {\tilde{\chi }} _{2}^{0}$, with a resulting signature of two ${\text {W}}$ bosons, two ${\text {Z}}$ bosons, or one of each (the case shown in Fig. 2e), and four light-flavor jets. This model, $\mathrm {T}5{{\text {qqqqWZ}}}$, with a resulting signature of one ${\text {W}}$ boson and one ${\text {Z}}$ boson, is studied with two different assumptions for the chargino mass: $m_{{\tilde{\chi }}^\pm _{1}} = 0.5(m_{{\tilde{\text {g}}}} + m_{{\tilde{\chi }}^{0}_{1}})$, and $m_{{\tilde{\chi }}^\pm _{1}} = m_{{\tilde{\chi }}^{0}_{1}}+20 \,{\text {GeV}}$, producing on- and off-shell bosons, respectively. The model is also considered with the assumption of decays to two ${\text {W}}$ bosons exclusively ($\mathrm {T}5{{\text {q q q qWW}}}$).

Figure 2a shows a model of bottom squark production with subsequent decay of ${\tilde{\text {b}}} _{1}\rightarrow {\text {t}}{\tilde{\chi }}^\pm _{1}$, yielding two ${\text {b}}$ quarks and four ${\text {W}}$ bosons. This model, $\mathrm {T}6{{\text {ttWW}}}$, is considered as a function of the the lightest bottom squark, ${\tilde{\text {b}}} _{1}$, and ${\tilde{\chi }}^\pm _{1}$ masses. The ${\tilde{\chi }}^{0}_{1}$ mass is fixed to be $50\,{\text {GeV}}$, causing two of the ${\text {W}}$ bosons to be produced off-shell when the ${\tilde{\chi }}^\pm _{1}$ mass is less than approximately $130\,{\text {GeV}}$. Figure 2b displays a model similar to $\mathrm {T}6{{\text{ttWW}}}$, but with top squark pair production and a subsequent decay of ${\tilde{\text {t}}} _{2}\rightarrow {\tilde{\text {t}}} _{1}{\text {H}}/{\text {Z}}$, with ${\tilde{\text {t}}} _{1}\rightarrow {\text {t}}{\tilde{\chi }}^{0}_{1}$, producing signatures with two ${\text {H}}$ bosons, two ${\text {Z}}$ bosons, or one of each. In this model, $\mathrm {T}6{{\text{ttHZ}}}$, the ${\tilde{\chi }}^{0}_{1}$ mass is fixed such that $m({\tilde{\text {t}}} _{1})-m({\tilde{\chi }}^{0}_{1})=m_{{\text {t}}}$.

The R parity violating decays considered in this analysis are $\mathrm {T}1{{\text{qqqqL}}}$ (Fig. 3a) and $\mathrm {T}1{{\text {tbs}}}$ (Fig. 3b). In $\mathrm {T}1{{\text {qqqqL}}}$, the gluino decays to the lightest squark (${\tilde{\text {g}}} \rightarrow {\tilde{\text {q}}} {\text {q}}$), which in turn decays to a quark (${\tilde{\text {q}}} \rightarrow {\text {q}}{\tilde{\chi }}^{0}_{1}$), but decays with the ${\tilde{\chi }}^{0}_{1}$ off shell (violating R parity) into two quarks and a charged lepton, giving rise to a prompt 5-body decay of the gluino. In $\mathrm {T}1{{\text{tbs}}}$, each gluino decays into three different SM quarks (a top, a bottom, and a strange quark).

## The CMS detector and event reconstruction

The central feature of the CMS detector is a superconducting solenoid of $6\,{\text {m}}$ internal diameter, providing a magnetic field of $3.8\,{\text {T}}$. Within the solenoid volume are a silicon pixel and strip tracker, a lead tungstate crystal electromagnetic calorimeter (ECAL), and a brass and scintillator hadron calorimeter (HCAL), each composed of a barrel and two endcap sections. Forward calorimeters extend the pseudorapidity ($\eta$) coverage provided by the barrel and endcap detectors. Muons are detected in gas-ionization chambers embedded in the steel flux-return yoke outside the solenoid. A more detailed description of the CMS detector, together with a definition of the coordinate system used and the relevant kinematic variables, can be found in Ref. [59].

Events of interest are selected using a two-tiered trigger system [60]. The first level, composed of custom hardware processors, uses information from the calorimeters and muon detectors to select events at a rate of around $100\,{\text {kHz}}$ within a fixed time interval of less than 4 μs. The second level, known as the high-level trigger, consists of a farm of processors running a version of the full event reconstruction software optimized for fast processing, and reduces the event rate to around $1\,{\text {kHz}}$ before data storage.

The reconstructed vertex with the largest value of summed physics-object squared-transverse-momentum is taken to be the primary ${\text {pp}}$ interaction vertex. The physics objects are the jets, clustered using the jet finding algorithm of Refs. [61, 62] with the tracks assigned to the vertex as inputs, and the associated missing transverse momentum, taken as the negative vector sum of the transverse momentum ($p_{{\mathrm {T}}}$) of those jets.

The particle-flow (PF) algorithm [63] aims to reconstruct and identify each individual particle in an event, with an optimized combination of information from the various elements of the CMS detector. The energy of photons is directly obtained from the ECAL measurement. The energy of electrons is determined from a combination of the electron momentum at the primary interaction vertex as determined by the tracker, the energy of the corresponding ECAL cluster, and the energy sum of all bremsstrahlung photons spatially compatible with the electron track [64]. The momentum of muons is obtained from the curvature of the corresponding track, combining information from the silicon tracker and the muon system [65]. The energy of charged hadrons is determined from a combination of their momentum measured in the tracker and the matching ECAL and HCAL energy deposits, corrected for the response function of the calorimeters to hadronic showers. The energy of neutral hadrons is obtained from the corresponding corrected ECAL and HCAL energies.

Hadronic jets are clustered from charged PF candidates associated with the primary vertex and from all neutral PF candidates using the anti-$k_{{\mathrm {T}}}$ algorithm [61, 62] with a distance parameter of 0.4. The jet momentum is determined as the vectorial sum of all PF candidate momenta in the jet. An offset correction is applied to jet energies to take into account the contribution from pileup [66]. Additional jet energy corrections are derived from simulation to bring the detector response to unity, and are improved with in situ measurements of the energy balance in dijet, multijet, photon+jets, and leptonically decaying ${\text {Z+jets}}$ events [67, 68]. Additional selection criteria are applied to each jet to remove jets potentially dominated by instrumental effects or reconstruction failures. Jets originating from ${\text {b}}$ quarks are identified as ${\text {b}}$-tagged jets using a deep neural network algorithm, DeepCSV [69], with a working point chosen such that the efficiency to identify a ${\text {b}}$ jet is 55–70% for a jet $p_{{\mathrm {T}}}$ between 20 and $400\,{\text {GeV}}$. The misidentification rate for a light-flavor jet is 1–2% in the same jet $p_{{\mathrm {T}}}$ range.

The vector ${\vec {p}}_{{\mathrm {T}}}^{{\text {miss}}}$ is defined as the projection onto the plane perpendicular to the beams of the negative vector sum of the momenta of all reconstructed PF candidates in an event [70]. Its magnitude, called missing transverse momentum, is referred to as $p_{{\mathrm {T}}} ^{\text {miss}}$. The scalar $p_{{\mathrm {T}}}$ sum of all jets in an event is referred to as $H_{\text {T}}$.

## Search strategy and event selection

The search strategy is similar to the one adopted in Refs. [24, 25]. The event selection requires the presence of at least two hadronic jets and at least two leptons, among which is an SS pair, as described below. Each selected event is assigned to an SR, based on its content. Maximum likelihood fits of the background (or signal plus background) predictions to the data in all SRs are then performed. Such a strategy ensures sensitivity to a broad range of possible signatures of new physics, even beyond the signal benchmarks considered in this analysis.

The kinematic requirements applied to leptons and jets are presented in Table 1. The analysis requires at least two jets with $p_{{\mathrm {T}}} >40\,{\text {GeV}}$ and two light SS leptons with $p_{{\mathrm {T}}} >15\,{\text {GeV}}$ ($10\,{\text {GeV}}$) for electrons (muons). Electrons are identified based on a discriminant using shower shape and track quality variables, while the muon identification relies on the quality of the geometrical matching between the tracker and muon system measurements. In order to reject leptons from the decay of heavy flavor hadrons, the tracks are required to have an impact parameter compatible with the position of the primary vertex. Several isolation criteria are also applied, based on the scalar sum of hadron and photon $p_{{\mathrm {T}}}$ within a cone centered on the lepton direction and whose radius decreases with its $p_{{\mathrm {T}}}$, the ratio of the $p_{{\mathrm {T}}}$ of the lepton to that of the closest jet, and the relative $p_{{\mathrm {T}}}$ of the lepton to that of the closest jet after lepton momentum subtraction. These criteria are designed to mitigate the loss of lepton efficiency caused by lepton-jet overlaps that occurs frequently in events with significant hadronic activity. A more detailed description of the set of identification and isolation variables used in the lepton selection can be found in Ref. [71].

The lepton reconstruction and identification efficiency is in the range of 45–70% (70–90%) for electrons (muons), with $p_{{\mathrm {T}}} >25\,{\text {GeV}}$, increasing as a function of $p_{{\mathrm {T}}}$ and reaching the maximum value for $p_{{\mathrm {T}}} >60\,{\text {GeV}}$. In the low-momentum regime, $15<p_{{\mathrm {T}}} <25\,{\text {GeV}}$ for electrons and $10<p_{{\mathrm {T}}} <25\,{\text {GeV}}$ for muons, the efficiencies are approximately 40% for electrons and 55% for muons. The lepton trigger efficiency for electrons is in the range of 90–98%, converging to the maximum value for $p_{{\mathrm {T}}} >30 \,{\text {GeV}}$, and it is around 92% for muons.

**Table 1:** Transverse momentum and pseudorapidity requirements for leptons and jets. Note that the $p_{{\mathrm {T}}}$ thresholds to count jets and ${\text {b}}$-tagged jets are different; the jet multiplicity $N_\text {jets}$ includes ${\text {b}}$-tagged jets if their $p_{{\mathrm {T}}}$ exceeds $40\,{\text {GeV}}$

Object	$p_{{\mathrm {T}}}$ (${\text {GeV}}$)	$|\eta |$
Electrons	$>15$	$<2.5$
Muons	$>10$	$<2.4$
Jets	$>40$	$<2.4$
${\text {b}}$-tagged jets	$>25$	$<2.4$

In order to reduce backgrounds from the decays of ${\text {c}}$- and ${\text {b}}$-hadrons or from the Drell–Yan process, we reject events with same-flavor lepton pairs with invariant mass ($m_{\ell \ell }$) less than $12\,{\text {GeV}}$, where leptons are reconstructed with a looser set of requirements compared to the nominal selection. Furthermore, events containing a lepton pair with $m_{\ell \ell }< 8 \,{\text {GeV}}$, regardless of charge or flavor, are rejected in order to emulate a similar condition applied at the trigger level. Events are then separated according to the $p_{{\mathrm {T}}}$ of the leptons forming the SS pair: high-high if both have $p_{{\mathrm {T}}} > 25\,{\text {GeV}}$, low-low if both have $p_{{\mathrm {T}}} < 25\,{\text {GeV}}$, and high-low otherwise.

Two sets of trigger algorithms are used to select the events: pure dilepton triggers, which require the presence of two isolated leptons with $p_{{\mathrm {T}}}$ thresholds on the leading (subleading) lepton in the 17–23 (8–12) $\,{\text {GeV}}$ range, and dilepton triggers with no isolation requirements, a lower $p_{{\mathrm {T}}}$ threshold of $8\,{\text {GeV}}$, an invariant mass condition $m_{\ell \ell }> 8 \,{\text {GeV}}$ to reject low mass resonances, and with a minimum $H_{\text {T}}$ in the range of $300{-}350\,{\text {GeV}}$. The ranges listed here reflect the varying trigger conditions during the data taking periods. The pure dilepton triggers are used to select high-high and high-low pairs, while low-low pairs are selected using the triggers with $H_{\text {T}}$ requirements.

Six exclusive categories are then defined as follows:

- High-High SS pair, significant $p_{{\mathrm {T}}} ^{\text {miss}}$ (HH): exactly 2 leptons, both with $p_{{\mathrm {T}}} >25 \,{\text {GeV}}$, and $p_{{\mathrm {T}}} ^{\text {miss}} >50 \,{\text {GeV}}$;
- High-Low SS pair, significant $p_{{\mathrm {T}}} ^{\text {miss}}$ (HL): exactly 2 leptons, one with $p_{{\mathrm {T}}} >25 \,{\text {GeV}}$, one with $p_{{\mathrm {T}}} <25 \,{\text {GeV}}$, and $p_{{\mathrm {T}}} ^{\text {miss}} >50 \,{\text {GeV}}$;
- Low-Low SS pair, significant $p_{{\mathrm {T}}} ^{\text {miss}}$ (LL): exactly 2 leptons, both with $p_{{\mathrm {T}}} <25 \,{\text {GeV}}$ and $p_{{\mathrm {T}}} ^{\text {miss}} >50 \,{\text {GeV}}$;
- Low $p_{{\mathrm {T}}} ^{\text {miss}}$ (LM): exactly 2 leptons, both with $p_{{\mathrm {T}}} >25 \,{\text {GeV}}$, and $p_{{\mathrm {T}}} ^{\text {miss}} <50 \,{\text {GeV}}$; and
- Multilepton with an on-shell Z boson (on-${\text {Z}}$ ML): $\ge$3 leptons, at least one with $p_{{\mathrm {T}}} >25 \,{\text {GeV}}$, $p_{{\mathrm {T}}} ^{\text {miss}} >50 \,{\text {GeV}}$, $\ge {\text {Z}}$ boson candidate formed by a pair of opposite-sign (OS), same-flavor leptons with $76< m_{\ell \ell }< 106 \,{\text {GeV}}$.
- Multilepton without an on-shell Z boson (off-${\text {Z}}$ ML): same as on-${\text {Z}}$ ML but without a ${\text {Z}}$ boson candidate.

The categories are typically sensitive to different new physics scenarios and enriched in different SM backgrounds. For example the HH category drives the sensitivity for most of the RPC scenarios ($\mathrm {T}1{{\text {tttt}}}$, $\mathrm {T}5{{\text {ttbbWW}}}$, $\mathrm {T}5{{\text {tttt}}}$, $\mathrm {T}1{{\text {tttt}}}$, $\mathrm {T}5{{\text {qqqqWW}}}$) with a large mass splitting. The HL and LL categories become relevant for lower mass splitting when one or both leptons tend to be soft. Scenarios resulting in the presence of one or multiple ${\text {Z}}$ bosons in the final state such as $\mathrm {T}5{{\text {q}} {\text {qqqWZ}}}$ and $\mathrm {T}6{{\text {ttHZ}}}$ will typically be primarily constrained by the on-Z or off-Z category, also depending on the considered SUSY mass spectrum. Finally the LM category enhances the analysis sensitivity for RPV scenarios, in particular for $\mathrm {T}1{{\text {qqqqL}}}$ where no genuine $p_{{\mathrm {T}}} ^{\text {miss}}$ is expected.

Various SRs are constructed based on the jet multiplicity $N_\text {jets}$, the ${\text {b}}$-tagged jet multiplicity $N_{{\text {b}}}$, $H_{\text {T}}$, $p_{{\mathrm {T}}} ^{\text {miss}}$, the charge of the SS pair, and $m_{\text {T}} ^{\text {min}}$, which is defined below. The $m_{\text {T}} ^{\text {min}}$ variable, introduced in Ref. [71], is defined as the minimum of the transverse masses calculated from each of the leptons forming the SS pair and ${\vec {p}}_{{\mathrm {T}}}^{{\text {miss}}}$, except for the on-${\text {Z}}$ ML category where we only consider the transverse mass computed using the leptons not forming the ${\text {Z}}$ candidate. It is characterized by a kinematic cutoff for events where $p_{{\mathrm {T}}} ^{\text {miss}}$ only arises from the leptonic decay of a single ${\text {W}}$ boson and is effective at discriminating signal and background signatures.

A subset of SRs is split by the charge of the leptons in an SS pair which is used to take advantage of the charge asymmetry in most of the background processes, such as ${\text {W}}{\text {Z}}$, $\hbox {t}{\bar{\hbox {t}}} {\text {W}}$ or SS ${\text {W}}{\text {W}}$. The SRs corresponding to each category, HH, HL, LL, LM, on-${\text {Z}}$ ML, and off-${\text {Z}}$ ML, are summarized in Tables 2, 3, 4, 5, 6 and 7, respectively. The binning ranges are chosen to maximize the sensitivity to a variety of SUSY benchmark points and are such that the expected SM yield in any SR has relative statistical uncertainties typically smaller than unity.

**Table 2:** The SR definitions for the HH category. Charge-split regions are indicated with (++) and (- -). The three highest $H_{\text {T}}$ regions are split only by $N_\text {jets}$, resulting in 62 regions in total. Quantities are specified in units of $\,{\text {GeV}}$ where applicable

$N_{{\text {b}}}$	$m_{\text {T}} ^{\text {min}}$	$p_{{\mathrm {T}}} ^{\text {miss}}$	$N_\text {jets}$	$H_{\text {T}} < 300$	$H_{\text {T}} \in [300, 1125]$	$H_{\text {T}} \in [1125, 1300]$	$H_{\text {T}} \in [1300, 1600]$	$H_{\text {T}} > 1600$
0	${<}120$	50–200	2–4	SR1	SR2	SR54 $N_\text {jets} < 5$	SR55 $N_\text {jets} < 5$	SR56 $N_\text {jets} < 5$
			$\ge$5	SR3	SR4			
		200–300	2–4		SR5 (++)/SR6 (- -)			
			$\ge$5		SR7			
	${>}120$	50–200	2–4		SR8 (++)/SR9 (- -)			
			$\ge$5		SR10			
		200–300	2–4					
			$\ge$5					
1	${<}120$	50–200	2–4	SR11	SR12	SR57 $N_\text {jets} =$ 5 or 6	SR58 $N_\text {jets} =$ 5 or 6	SR59 $N_\text {jets} =$ 5 or 6
			$\ge$5	SR13 (++)/SR14 (- -)	SR15 (++)/SR16 (- -)			
		200–300	2–4		SR17 (++)/SR18 (- -)			
			$\ge$5		SR19			
	${>}120$	50–200	2–4		SR20 (++)/SR21 (- -)			
			$\ge$5		SR22			
		200–300	2–4					
			$\ge$5					
2	${<}120$	50–200	2–4	SR23	SR24	SR60 $N_\text {jets} > 6$	SR61 $N_\text {jets} > 6$	SR62 $N_\text {jets} > 6$
			$\ge$5	SR25 (++)/SR26 (- -)	SR27 (++)/SR28 (- -)			
		200–300	2–4		SR29 (++)/SR30 (- -)			
			$\ge$5		SR31			
	${>}120$	50–200	2–4		SR32 (++)/SR33 (- -)			
			$\ge$5		SR34			
		200–300	2–4					
			$\ge$5					
$\ge$3	${<}120$	50–200	2–4	SR35 (++)/SR36 (- -)	SR37 (++)/SR38 (- -)			
			$\ge$5		SR39 (++)/SR40 (- -)			
		200–300	2–4		SR37 (++)/SR38 (- -)			
			$\ge$5		SR39 (++)/SR40 (- -)			
	${>}120$	50–300	2–4	SR41	SR42 (++)/SR43 (- -)			
			$\ge$5		SR44 (++) / SR45 (- -)			
Inclusive	Inclusive	300–500	2–4	—	SR46 (++)/SR47 (- -)			
		${>}500$			SR48 (++)/SR49 (- -)			
		300–500	$\ge$5		SR50 (++)/SR51 (- -)			
		${>}500$			SR52 (++)/SR53 (- -)

**Table 3:** The SR definitions for the HL category. Charge-split regions are indicated with (++) and (- -). There are 43 regions in total. Quantities are specified in units of $\,{\text {GeV}}$ where applicable

$N_{{\text {b}}}$	$m_{\text {T}} ^{\text {min}}$	$p_{{\mathrm {T}}} ^{\text {miss}}$	$N_\text {jets}$	$H_{\text {T}} < 300$	$H_{\text {T}} \in [300, 1125]$	$H_{\text {T}} \in [1125, 1300]$	$H_{\text {T}} > 1300$
0	${<}120$	50–200	2–4	SR1	SR2	SR40 (++)/SR41 (- -)	SR42 (++)/SR43 (- -)
			$\ge$5	SR3	SR4		
		200–300	2–4		SR5 (++)/SR6 (- -)		
			$\ge$5		SR7		
1	${<}120$	50–200	2–4	SR8	SR9		
			$\ge$5	SR10 (++)/SR11 (- -)	SR12 (++) / SR13 (- -)		
		200–300	2–4		SR14		
			$\ge$5		SR15 (++)/SR16 (- -)		
2	${<}120$	50–200	2–4	SR17	SR18		
			$\ge$5	SR19 (++)/SR20 (- -)	SR21 (++) / SR22 (- -)		
		200–300	2–4		SR23 (++)/SR24 (- -)		
			$\ge$5		SR25		
$\ge$3	${<}120$	50–200	$\ge$2	SR26 (++)/SR27 (- -)	SR28 (++)/SR29 (- -)		
		200–300			SR30		
Inclusive	${>}120$	50–300	$\ge$2	SR31	SR32		
Inclusive	Inclusive	300–500	2–4	—	SR33 (++)/SR34 (- -)		
		${>}500$			SR35 (++)/SR36 (- -)		
		300–500	$\ge$5		SR37 (++)/SR38 (- -)		
		${>}500$			SR39

**Table 4:** The SR definitions for the LL category. All SRs in this category require $N_\text {jets} \ge 2$. There are 8 regions in total. Quantities are specified in units of $\,{\text {GeV}}$ where applicable

$N_{{\text {b}}}$	$m_{\text {T}} ^{\text {min}}$	$H_{\text {T}}$	$p_{{\mathrm {T}}} ^{\text {miss}} \in [50, 200]$	$p_{{\mathrm {T}}} ^{\text {miss}} > 200$
0	${<}120$	${>}400$	SR1	SR2
1			SR3	SR4
2			SR5	SR6
$\ge$3			SR7	
Inclusive	${>}120$		SR8

**Table 5:** The SR definitions for the LM category. All SRs in this category require $p_{{\mathrm {T}}} ^{\text {miss}} < 50\,{\text {GeV}}$ and $H_{\text {T}} >300\,{\text {GeV}}$. The two high-$H_{\text {T}}$ regions are split only by $N_\text {jets}$, resulting in 11 regions in total. Quantities are specified in units of $\,{\text {GeV}}$ where applicable

$N_{{\text {b}}}$	$N_\text {jets}$	$H_{\text {T}} \in [300,1125]$	$H_{\text {T}} \in [1125,1300]$	$H_{\text {T}} > 1300$
0	2–4	SR1	SR8 ($N_\text {jets} <5$)	SR10 ($N_\text {jets} <5$)
	$\ge$5	SR2		
1	2–4	SR3		
	$\ge$5	SR4	SR9 ($N_\text {jets} \ge 5$)	SR11 ($N_\text {jets} \ge 5$)
2	2–4	SR5		
	$\ge$5	SR6		
$\ge$3	$\ge$2	SR7

**Table 6:** The SR definitions for the on-${\text {Z}}$ ML category. All SRs in these categories require $N_\text {jets} \ge 2$. Regions marked with ${}^\dagger$ are split by $m_{\text {T}} ^{\text {min}} =120\,{\text {GeV}}$, with the high-$m_{\text {T}} ^{\text {min}}$ region specified by the second SR label. There are 23 regions in total. Quantities are specified in units of $\,{\text {GeV}}$ where applicable

$N_{{\text {b}}}$	$H_{\text {T}}$	$p_{{\mathrm {T}}} ^{\text {miss}} \in [50,150]$	$p_{{\mathrm {T}}} ^{\text {miss}} \in [150,300]$	$p_{{\mathrm {T}}} ^{\text {miss}} \ge 300$
0	${<}400$	SR1/SR2${}^\dagger$	SR3/SR4${}^\dagger$	SR22/SR23${}^\dagger$
	400–600	SR5/SR6${}^\dagger$	SR7/SR8${}^\dagger$	
1	${<}400$	SR9	SR10	
	400–600	SR11	SR12	
2	${<}400$	SR13	SR14	
	400–600	SR15	SR16	
$\ge$3	${<}600$	SR17		
Inclusive	$\ge$600	SR18/SR19${}^\dagger$	SR20/SR21${}^\dagger$

**Table 7:** The SR definitions for the off-${\text {Z}}$ category. All SRs in these categories require $N_\text {jets} \ge 2$. Regions marked with ${}^\dagger$ are split by $m_{\text {T}} ^{\text {min}} =120\,{\text {GeV}}$, with the high-$m_{\text {T}} ^{\text {min}}$ region specified by the second SR label. There are 21 regions in total. Quantities are specified in units of $\,{\text {GeV}}$ where applicable

$N_{{\text {b}}}$	$H_{\text {T}}$	$p_{{\mathrm {T}}} ^{\text {miss}} \in [50,150]$	$p_{{\mathrm {T}}} ^{\text {miss}} \in [150,300]$	$p_{{\mathrm {T}}} ^{\text {miss}} \ge 300$
0	${<}400$	SR1/SR2${}^\dagger$	SR3/SR4${}^\dagger$	SR20/SR21${}^\dagger$
	400–600	SR5	SR6	
1	${<}400$	SR7	SR8	
	400–600	SR9	SR10	
2	${<}400$	SR11	SR12	
	400–600	SR13	SR14	
$\ge$3	$<600$	SR15		
Inclusive	$\ge$600	SR16/SR17${}^\dagger$	SR18/SR19${}^\dagger$	

## Backgrounds

Several SM processes can lead to the signatures studied in this analysis. There are three background categories, depending on the lepton content of the event:

- Events with two or more prompt leptons, including an SS pair;
- Events with at least one nonprompt lepton (defined below); and
- Events with a pair of OS leptons, one of which is reconstructed with the wrong charge.

The first category includes a variety of low cross section processes where multiple electroweak bosons are produced, possibly in the decay of top quarks, which then decay leptonically leading to an SS lepton pair. This category usually dominates the background yields in SRs with large $p_{{\mathrm {T}}} ^{\text {miss}}$ or $H_{\text {T}}$ and in most of the ML SRs with a ${\text {Z}}$ candidate. The main contributions arise from the production of a ${\text {W}}{\text {Z}}$ or an SS ${\text {W}}$pair, or of a $\hbox {t}{\bar{\hbox {t}}}$ pair in association with a ${\text {W}}$, ${\text {Z}}$ or ${\text {H}}$ boson. The event yields for these processes are estimated individually. In contrast, the expected event yields from other rare processes (including ${\text {Z}}{\text {Z}}$, triple boson production, ${\text {tWZ}}$, ${\text {tZq}}$, $\hbox {t}{\bar{\hbox {t}}} \hbox {t}{\bar{\hbox {t}}}$, and double parton scattering) are summed up into a single contribution denoted as “Rare”. Processes including a genuine photon, such as ${\text {W}}{\upgamma }$, ${\text {Z}}{\upgamma }$, $\hbox {t}{\bar{\hbox {t}}} {\upgamma }$, and ${\text {t}}{\upgamma }$, are also considered and grouped together. They are referred to as “X${\upgamma }$”. All contributions from this category are estimated using simulated samples. Correction factors are applied to take into account small differences between data and simulation, including trigger, lepton selection, and ${\text {b}}$ tagging efficiencies, with associated systematic uncertainties listed in Sect. 6.

The second category consists of events where one of the selected leptons, generically denoted as “nonprompt lepton”, is either a decay product of a heavy flavor hadron or, more rarely, a misidentified hadron. This category is typically the dominant one in SRs with moderate or low $p_{{\mathrm {T}}} ^{\text {miss}}$ or low $m_{\text {T}} ^{\text {min}}$ (except for the on-${\text {Z}}$ ML SRs). This background is estimated directly from data using the “tight-to-loose” method [24, 25]. This method is based on the probability for a nonprompt lepton passing loose selection criteria to also satisfy the tighter lepton selection used in the analysis. The number of events in an SR with *N* leptons, including at least one nonprompt lepton, can be estimated by applying this probability to a corresponding control region (CR) of events with *N* loose leptons where at least one of them fails the tight selection.

The measurement of the tight-to-loose ratio is performed in a sample enriched in dijet events with exactly one loose lepton, low $p_{{\mathrm {T}}} ^{\text {miss}}$, and low $m_{\text {T}} ^{\text {min}}$. This sample is contaminated by prompt leptons from ${\text {W}}$ boson decays. The contamination is estimated from the $m_{\text {T}} ^{\text {min}}$ distribution, and it is subtracted before calculating the ratio. The tight-to-loose ratio is computed separately for electrons and muons, and is parameterized as a function of the lepton $\eta$ and $p_{{\mathrm {T}}} ^\text {corr}$. The $p_{{\mathrm {T}}} ^\text {corr}$ variable is defined as the sum of the lepton $p_{{\mathrm {T}}}$ and the energy in the isolation cone exceeding the isolation threshold value applied to tight leptons. This parametrization improves the stability of the tight-to-loose ratio with respect to variations in the $p_{{\mathrm {T}}}$ of the partons from which the leptons originate.

The performance of the tight-to-loose ratio was assessed in a MC closure test. A tight-to-loose ratio was extracted from a MC sample of QCD events. This ratio was then used to predict the number of events with one prompt and one nonprompt SS dileptons in MC $\hbox {t}{\bar{\hbox {t}}}$ and ${\text {W+jets}}$ events. The predicted and observed rates of SS dileptons were compared as a function of kinematic properties and found to agree within 30%. The data driven estimate was also compared to a direct prediction from simulation and a similar level of agreement was reached.

The final category is a subdominant background in all SRs and corresponds to events where the charge of a lepton is incorrectly measured. Charge misidentification primarily occurs when an electron undergoes bremsstrahlung in the tracker material or in the beam pipe. Similarly to the tight-to-loose method, the number of SS lepton pairs where one of the leptons has its charge misidentified can be determined using the number of OS pairs and the knowledge of the charge misidentification rate. We use simulation to parameterize this rate as a function of $p_{{\mathrm {T}}}$ and $\eta$ for electrons and find values varying between $10^{-5}$ (central electrons with $p_{{\mathrm {T}}} \approx 20\,{\text {GeV}}$) and $5\times 10^{-3}$ (forward electrons with $p_{{\mathrm {T}}} \approx 200\,{\text {GeV}}$). To calibrate the charge misidentification rate, we exploit the fact that charge misidentification only has a small effect on the electron energy measurement in the calorimeter. As a result, electron pairs from ${\text {Z}}$ boson decays yield a sharp peak near the ${\text {Z}}$ mass even when one of the electrons has a misidentified charge. The SS dielectron invariant mass distributions in data and MC can then be used to derive a correction factor to the MC charge misidentification rate. Good agreement between data and MC is found in 2016, while the charge misidentification rate in simulation corresponding to 2017 and 2018 data needs to be scaled up by a factor of 1.4. Muon charge misidentification arises from a relatively large uncertainty in the transverse momentum at high momentum or from a poor quality track. The various criteria applied in this analysis on the quality of the muon reconstruction lead to a misidentification rate at least one order of magnitude smaller than for electrons according to simulation. The muon charge misassignment has also been studied using cosmic ray muons with $p_{{\mathrm {T}}}$ up to several hundred $\,{\text {GeV}}$, confirming the predictions from simulation [72]. It is therefore neglected. Correction factors are however applied to the simulation to account for a possible difference in the selection efficiency related to these criteria.

## Systematic uncertainties

The predicted yields of signal and background processes are affected by several sources of uncertainty, summarized in Table 8. Depending on their source, they are treated as fully correlated or uncorrelated between the three years of data taking. Signal and background contributions estimated from simulation are affected by experimental uncertainties in the efficiency of the trigger, lepton reconstruction and identification [64, 73], the efficiency of ${\text {b}}$ tagging [69], the jet energy scale [67], the integrated luminosity [74–76]. An uncertainty is also assigned to the value of the inelastic cross section, which affects the pileup rate [77] and that can impact the description of the jet multiplicity or the $p_{{\mathrm {T}}} ^{\text {miss}}$ resolution. Simulation is also affected by theoretical uncertainties, which are evaluated by varying the factorization and renormalization scales up and down by a factor of two, and by using different PDFs within the NNPDF3.0 and 3.1 PDF sets [35, 36, 78]. These uncertainties can affect both the overall yield (normalization) and the relative population (shape) across the SRs. Background normalization uncertainties are increased to 30%, either to account for the additional hadronic activity required (for ${\text {W}}{\text {Z}}$ and ${\text {W}}^{\pm } {\text {W}}^{\pm }$) or to take into consideration recent measurements (for $\hbox {t}{\bar{\hbox {t}}} {\text {W}}$, $\hbox {t}{\bar{\hbox {t}}} {\text {Z}}$) [79, 80]. The Rare and X${\upgamma }$backgrounds, which are less well understood experimentally and theoretically, are assigned a 50% uncertainty.

To account for possible mismodeling of the flavor of additional jets, an additional 70% uncertainty is applied to $\hbox {t}{\bar{\hbox {t}}} {\text {W}}$, $\hbox {t}{\bar{\hbox {t}}} {\text {Z}}$, and $\hbox {t}{\bar{\hbox {t}}} {\text {H}}$ events produced in association with a pair of ${\text {b}}$ jets, reflecting the measured ratio of $\hbox {t}{\bar{\hbox {t}}} {\text {b}}{\overline{\text {b}}} / \hbox {t}{\bar{\hbox {t}}} \text {jj}$ cross sections reported in Ref. [81].

As discussed in Sect. 5, the nonprompt lepton and charge misidentification backgrounds are estimated from CRs. The associated uncertainties include the statistical uncertainties in the CR yields, as well as the systematic uncertainties in the extrapolations from the CRs to the SRs, as described below. In the case of the nonprompt lepton background, we include a 30% uncertainty from studies of the closure of the method in simulation. Furthermore, the uncertainty in the measurement of the tight-to-loose ratio, because of the prompt lepton contamination, results in a 1–30% additional uncertainty in the background yields. The charge misidentification background is assigned a 20% uncertainty based on a comparison of the kinematic properties of simulated and data events in the ${\text {Z}}\rightarrow {\text {e}}^{+} {{\text {e}}}^{-}$ CR with one electron or positron having a misidentified charge.

In general, the systematic uncertainties with the largest impact on the expected limits defined below are related to the lepton identification and isolation scale factors, the cross section of the rare processes, and the ${\text {WZ}}$ background normalization.

**Table 8:** Summary of the sources of systematic uncertainty and their effect on the yields of different processes in the SRs. The first two groups list experimental and theoretical uncertainties assigned to processes estimated using simulation, while the last group lists uncertainties assigned to processes whose yield is estimated from the data. The uncertainties in the first group also apply to signal samples. Reported values are representative for the most relevant signal regions

Source	Typical uncertainty (%)	Correlation across years
Integrated luminosity	2.3–2.5	Uncorrelated
Lepton selection	2–10	Uncorrelated
Trigger efficiency	2–7	Uncorrelated
Pileup	0–6	Uncorrelated
Jet energy scale	1–15	Uncorrelated
${\text {b}}$ tagging	1–10	Uncorrelated
Simulated sample size	1–20	Uncorrelated
Scale and PDF variations	10–20	Correlated
Theoretical background cross sections	30–50	Correlated
Nonprompt leptons	30	Correlated
Charge misidentification	20	Uncorrelated
$N_J^\text {ISR}$	1–30	Uncorrelated

## Results and interpretation

**Fig. 4:**
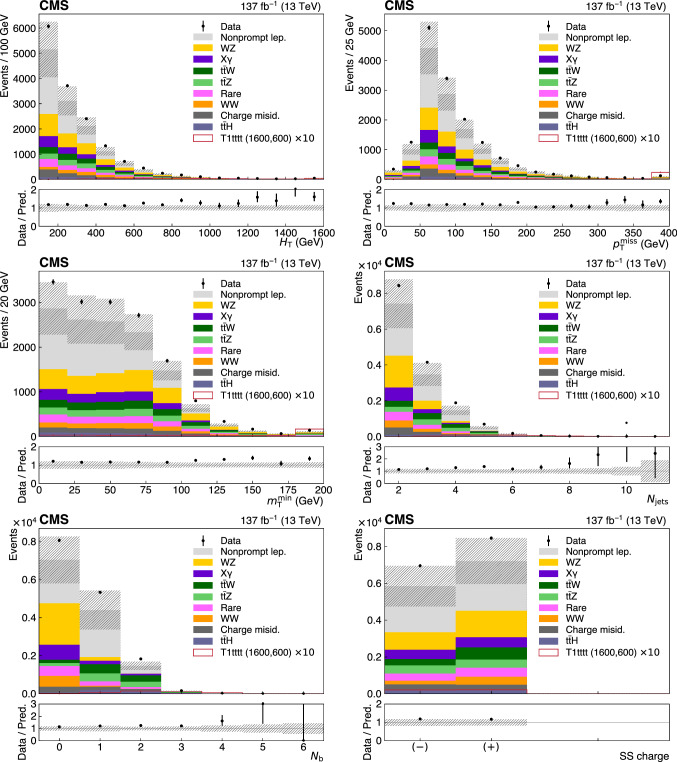
Distributions of the main analysis variables after the event selection: $H_{\text {T}}$, $p_{{\mathrm {T}}} ^{\text {miss}}$, $m_{\text {T}} ^{\text {min}}$, $N_\text {jets}$, $N_{{\text {b}}}$, and the charge of the SS pair, where the last bin includes the overflow (where applicable). The hatched area represents the total statistical and systematic uncertainty in the background prediction. The lower panels show the ratio of the observed event yield to the background prediction. The prediction for the SUSY model $\mathrm {T}1{{\text {tttt}}}$ with $m_{{\tilde{\text {g}}}}=1600\,{\text {GeV}}$ and $m_{{\tilde{\chi }}^{0}_{1}}=600\,{\text {GeV}}$ is overlaid

**Fig. 5:**
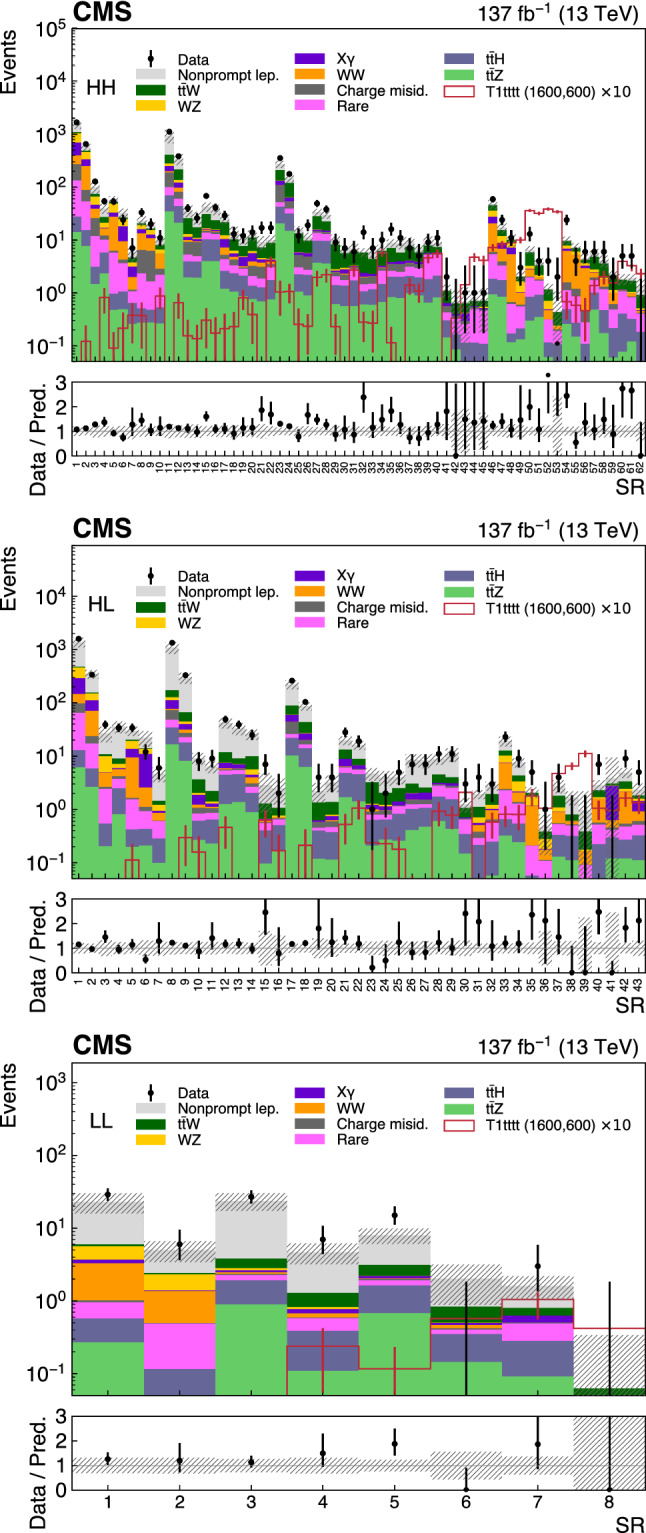
Expected and observed SR yields for the HH, HL, LL signal categories. The hatched area represents the total statistical and systematic uncertainty in the background prediction

**Fig. 6:**
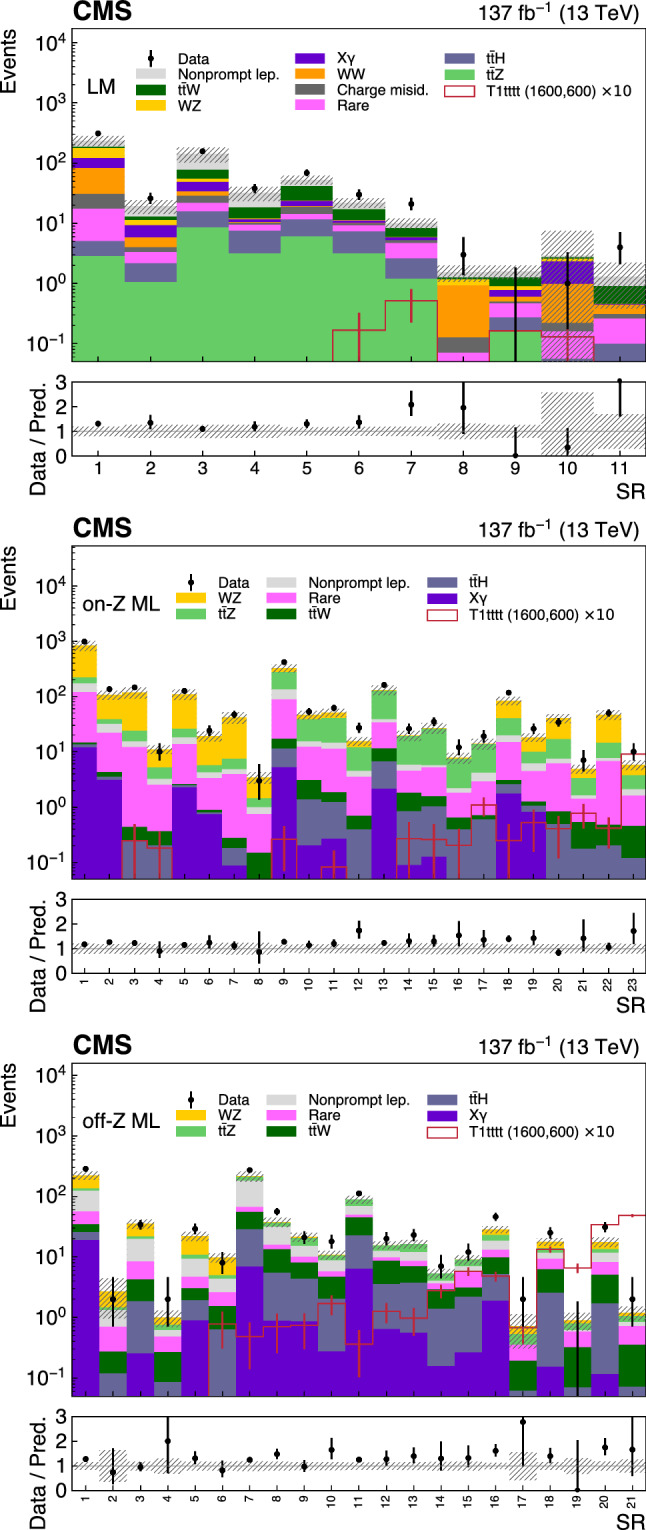
Expected and observed SR yields for the LM, on-${\text {Z}}$ ML, off-${\text {Z}}$ ML signal categories. The hatched area represents the total statistical and systematic uncertainty in the background prediction

The distributions of the variables used to define the SRs after the event selection are shown in Fig. 4. Background yields shown as stacked histograms in Figs. 4, 5, and 6 are those determined following the prescriptions detailed in Sect. 5. The overall data yields exceed expectation by an amount close to the systematic uncertainty. However, no particular trend that is not covered by the uncertainties discussed in the previous sections, is seen in the distributions. The significance of the excess is of similar magnitude in all categories, with a maximum of around 2 standard deviations (s.d.) in the off-${\text {Z}}$ ML category.

The results of the search, broken down by SR, are presented in Figs. 5 and 6, and are summarized in Table 9. No significant deviation with respect to the SM background prediction is observed. The largest excess of events found by fitting the data with the background-only hypothesis is in HH SR54, corresponding to a local significance of 2.6 s.d. Its neighboring bin, HH SR55, which is adjacent along the $H_{\text {T}}$ dimension, has a deficit of events in the data corresponding to a significance of 1.8 s.d.

These results are then interpreted as experimental constraints on the cross sections for the signal models discussed in Sect. 2. For each model, event yields in all SRs are used to obtain exclusion limits on the production cross section at 95% confidence level (${\text {CL}}$) with an asymptotic formulation of the modified frequentist $\text {CL}_\text {s}$ criterion [82–85], where uncertainties are incorporated as nuisance parameters and profiled [84]. This procedure takes advantage of the differences in the distribution of events amongst the SR between the various SM backgrounds and the signal considered. The normalizations of the various backgrounds are in particular allowed to float within their uncertainties in the global fit, resulting in several backgrounds (nonprompt lepton, $\hbox {t}{\bar{\hbox {t}}} {\text {W}}/{\text {Z}}/{\text {H}}$ and rare processes) being pulled up by around 1 s.d. for most of the signal points considered, which are often characterized by a distinctive distribution of events across the SRs. This observation is consistent with the current measurements of $\hbox {t}{\bar{\hbox {t}}} {\text {W}}$ and $\hbox {t}{\bar{\hbox {t}}} {\text {Z}}$ processes performed by the ATLAS and CMS Collaborations [79, 80]. The limits obtained are then used together with the theoretical cross section calculations to exclude regions of SUSY parameter space.

**Table 9:** Expected background event yields, total uncertainties, and observed event yields in the SRs used in this search

HH regions			LL regions			Off-Z ML regions		
SR	Expected SM	Obs.	SR	Expected SM	Obs.	SR	Expected SM	Obs.
1	$1560\pm 300$	1673	1	$1390\pm 300$	1593	1	$235\pm 47$	309
2	$582\pm 93$	653	2	$348\pm 67$	337	2	$19.3\pm 5.2$	26
3	$100\pm 25$	128	3	$26.9\pm 8.8$	39	3	$142\pm 39$	156
4	$39.5\pm 8.5$	54	4	$35.9\pm 9.1$	34	4	$32.2\pm 8.8$	38
5	$57.7\pm 9.9$	53	5	$29.8\pm 6.0$	34	5	$53.0\pm 9.1$	69
6	$32.5\pm 7.1$	24	6	$22.2\pm 7.2$	12	6	$22.0\pm 4.0$	30
7	$5.5\pm 1.8$	7	7	$4.7\pm 1.4$	6	7	$10.1\pm 2.0$	21
8	$22.9\pm 5.1$	33	8	$1100\pm 280$	1342	8	$1.53\pm 0.48$	3
9	$19.5\pm 3.9$	20	9	$299\pm 71$	330	9	$1.58\pm 0.41$	0
10	$9.6\pm 1.9$	11	10	$9.1\pm 2.3$	8	10	$2.9\pm 2.9$	1
11	$940\pm 270$	1115	11	$6.4\pm 1.6$	9	11	$1.31\pm 0.93$	4
12	$340\pm 81$	384	12	$42.1\pm 9.2$	49			
13	$36.3\pm 9.5$	40	13	$33.0\pm 8.4$	39	On-${\text {Z}}$ ML regions		
14	$26.8\pm 7.4$	26	14	$25.8\pm 5.9$	25	SR	Expected SM	Obs.
15	$42.7\pm 8.6$	68	15	$2.8\pm 2.0$	7	1	$840\pm 170$	985
16	$37.9\pm 8.6$	41	16	$2.5\pm 1.3$	2	2	$107\pm 21$	136
17	$26.5\pm 6.2$	29	17	$222\pm 42$	260	3	$119\pm 27$	146
18	$14.3\pm 3.6$	13	18	$86\pm 15$	104	4	$11.1\pm 2.1$	10
19	$10.6\pm 2.5$	12	19	$2.22\pm 0.90$	4	5	$109\pm 24$	126
20	$12.3\pm 2.9$	14	20	$3.2\pm 1.1$	4	6	$19.3\pm 4.1$	24
21	$9.2\pm 2.7$	17	21	$19.8\pm 3.8$	28	7	$42\pm 10$	47
22	$10.1\pm 2.1$	17	22	$16.1\pm 3.0$	19	8	$3.47\pm 0.84$	3
23	$272\pm 43$	354	23	$4.7\pm 1.3$	1	9	$327\pm 54$	419
24	$147\pm 25$	177	24	$4.0\pm 1.2$	2	10	$46.5\pm 8.4$	53
25	$15.3\pm 2.9$	12	25	$4.0\pm 1.1$	5	11	$51.3\pm 9.1$	62
26	$11.4\pm 2.4$	19	26	$8.5\pm 2.4$	7	12	$15.6\pm 2.8$	27
27	$33.4\pm 5.4$	49	27	$8.4\pm 2.5$	7	13	$131\pm 27$	162
28	$30.1\pm 4.9$	38	28	$8.9\pm 2.2$	11	14	$19.9\pm 4.3$	26
29	$10.4\pm 2.2$	9	29	$10.9\pm 3.1$	11	15	$26.9\pm 6.1$	35
30	$6.6\pm 1.3$	7	30	$1.25\pm 0.39$	3	16	$7.8\pm 1.8$	12
31	$6.9\pm 1.5$	6	31	$1.92\pm 0.37$	4	17	$14.0\pm 3.1$	19
32	$5.9\pm 1.1$	14	32	$2.77\pm 0.56$	3	18	$84\pm 15$	117
33	$6.1\pm 1.6$	7	33	$19.1\pm 4.1$	23	19	$18.2\pm 3.3$	26
34	$6.8\pm 1.3$	10	34	$7.5\pm 1.5$	9	20	$40.4\pm 7.6$	34
35	$8.8\pm 1.5$	16	35	$2.12\pm 0.49$	5	21	$4.92\pm 0.88$	7
36	$8.7\pm 2.0$	11	36	$0.47\pm 0.33$	1	22	$46.9\pm 9.9$	50
37	$9.4\pm 1.9$	7	37	$2.75\pm 0.77$	4	23	$5.8\pm 1.2$	10
38	$7.0\pm 1.3$	5	38	$1.68\pm 0.50$	0			
39	$9.6\pm 2.1$	9	39	$0.97\pm 0.97$	0	Off-${\text {Z}}$ ML regions		
40	$8.6\pm 1.7$	11	40	$2.83\pm 0.70$	7	SR	Expected SM	Obs.
41	$1.10\pm 0.32$	2	41	$3.8\pm 3.8$	0	1	$222\pm 36$	285
42	$0.63\pm 0.49$	0	42	$4.9\pm 1.0$	9	2	$2.7\pm 1.7$	2
43	$0.67\pm 0.60$	1	43	$2.36\pm 0.72$	5	3	$35.5\pm 6.4$	34
44	$0.74\pm 0.27$	1				4	$0.99\pm 0.31$	2
45	$0.71\pm 0.53$	1	LL regions			5	$22.1\pm 4.0$	29
46	$47.8\pm 9.7$	59	SR	Expected SM	Obs.	6	$9.7\pm 1.7$	8
47	$17.3\pm 3.8$	24	1	$23.0\pm 7.2$	29	7	$217\pm 44$	272
48	$10.3\pm 2.9$	11	2	$5.0\pm 1.6$	6	8	$37.7\pm 6.8$	56
49	$2.06\pm 0.49$	3	3	$23.8\pm 6.6$	27	9	$21.4\pm 3.7$	21
50	$6.5\pm 1.1$	13	4	$4.7\pm 1.5$	7	10	$10.9\pm 1.9$	18
51	$3.72\pm 0.79$	4	5	$8.0\pm 1.9$	15	11	$89\pm 14$	112
52	$1.21\pm 0.29$	4	6	$2.0\pm 1.1$	0	12	$15.6\pm 2.4$	20
53	$0.44\pm 0.44$	2	7	$1.61\pm 0.59$	3	13	$16.4\pm 2.7$	23
54	$9.8\pm 1.8$	24	8	$0.06\pm 0.06$	0	14	$5.36\pm 0.95$	7
55	$7.3\pm 1.4$	4				15	$9.0\pm 1.6$	12
56	$4.44\pm 0.98$	6				16	$28.4\pm 3.9$	46
57	$5.7\pm 1.1$	6				17	$0.72\pm 0.41$	2
58	$4.0\pm 1.0$	6				18	$17.8\pm 2.8$	25
59	$2.24\pm 0.53$	2				19	$0.89\pm 0.29$	0
60	$1.83\pm 0.44$	5				20	$17.7\pm 3.3$	31
61	$1.88\pm 0.40$	5				21	$1.20\pm 0.32$	2
62	$1.35\pm 0.56$	0						

**Fig. 7:**
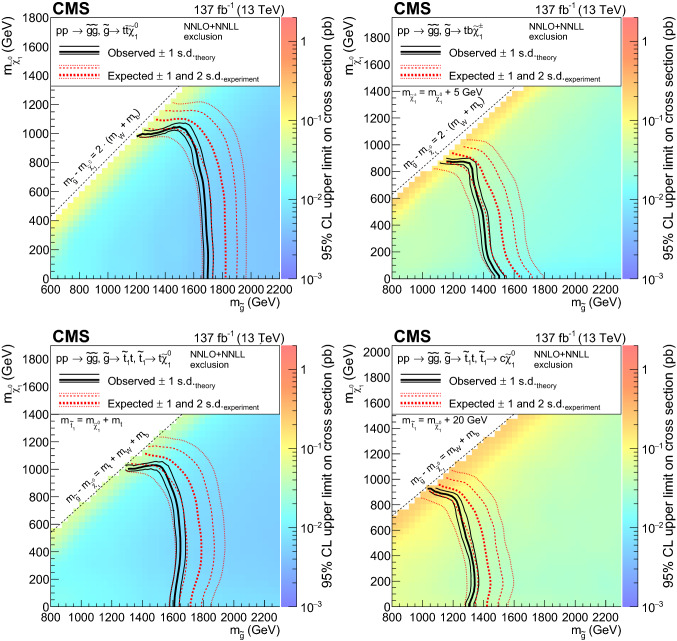
Exclusion regions at 95% ${\text {CL}}$ in the $m_{{\tilde{\chi }}^{0}_{1}}$ versus $m_{{\tilde{\text {g}}}}$ plane for the $\mathrm {T}1{{\text {tttt}}}$  (upper left) and $\mathrm {T}5{{\text {ttbbWW}}}$  (upper right) models, with off-shell third-generation squarks, and the $\mathrm {T}5{{\text {tttt}}}$  (lower left) and $\mathrm {T}5{{\text {ttcc}}}$ (lower right) models, with on-shell third-generation squarks. For the $\mathrm {T}5{{\text {ttbbWW}}}$ model, $m_{{\tilde{\chi }}^\pm _{1}} = m_{{\tilde{\chi }}^{0}_{1}} + 5\,{\text {GeV}}$, for the $\mathrm {T}5{{\text {tttt}}}$ model, $m_{{\tilde{\text {t}}}} - m_{{\tilde{\chi }}^{0}_{1}} = m_{{\text {t}}}$, and for the $\mathrm {T}5{{\text {ttcc}}}$ model, $m_{{\tilde{\text {t}}}} - m_{{\tilde{\chi }}^{0}_{1}} = 20\,{\text {GeV}}$ and the decay proceeds through ${\tilde{\text {t}}} \rightarrow {\text {c}}{\tilde{\chi }}^{0}_{1}$. The right-hand side color scale indicates the excluded cross section values for a given point in the SUSY particle mass plane. The solid black curves represent the observed exclusion limits assuming the approximate-NNLO+NNLL cross sections [46–51, 58] (thick line), or their variations of $\pm 1$ standard deviations (s.d.) (thin lines). The dashed red curves show the expected limits with the corresponding $\pm 1$ s.d. and $\pm 2$ s.d. uncertainties. Excluded regions are to the left and below the limit curves

**Fig. 8:**
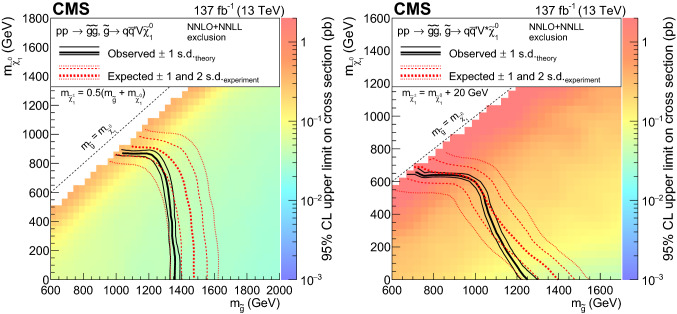
Exclusion regions at 95% ${\text {CL}}$ in the plane of $m_{{\tilde{\chi }}^{0}_{1}}$ versus $m_{{\tilde{\text {g}}}}$ for the $\mathrm {T}5{{\text {qqqqWZ}}}$ model with $m_{{\tilde{\chi }}^\pm _{1}}=0.5(m_{{\tilde{\text {g}}}} + m_{{\tilde{\chi }}^{0}_{1}})$ (left) and with $m_{{\tilde{\chi }}^\pm _{1}} = m_{{\tilde{\chi }}^{0}_{1}} + 20\,{\text {GeV}}$ (right). The notations are as in Fig. 7

**Fig. 9:**
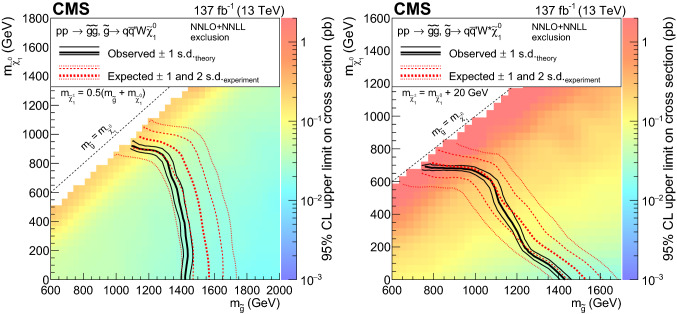
Exclusion regions at 95% ${\text {CL}}$ in the plane of $m_{{\tilde{\chi }}^{0}_{1}}$ versus $m_{{\tilde{\text {g}}}}$ for the $\mathrm {T}5{{\text {qqqqWW}}}$ model with $m_{{\tilde{\chi }}^\pm _{1}}=0.5(m_{{\tilde{\text {g}}}} + m_{{\tilde{\chi }}^{0}_{1}})$ (left) and with $m_{{\tilde{\chi }}^\pm _{1}} = m_{{\tilde{\chi }}^{0}_{1}} + 20\,{\text {GeV}}$ (right). The notations are as in Fig. 7

**Fig. 10:**
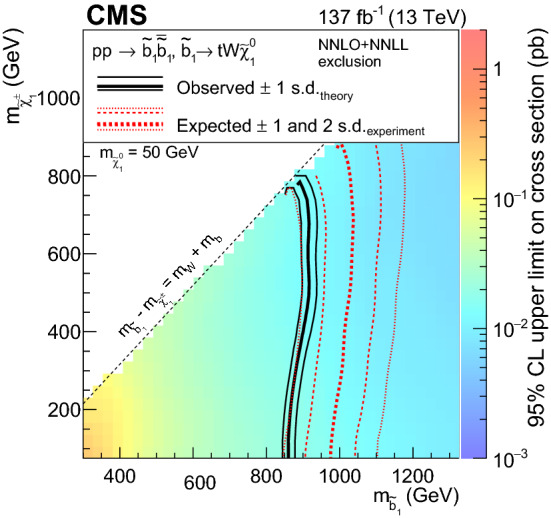
Exclusion regions at 95% ${\text {CL}}$ in the plane of $m_{{\tilde{\chi }}^\pm _{1}}$ versus $m_{{\tilde{\text {b}}} _{1}}$ for the $\mathrm {T}6{{\text {ttWW}}}$ model with $m_{{\tilde{\chi }}^{0}_{1}}=50\,{\text {GeV}}$. The notations are as in Fig. 7

**Fig. 11:**
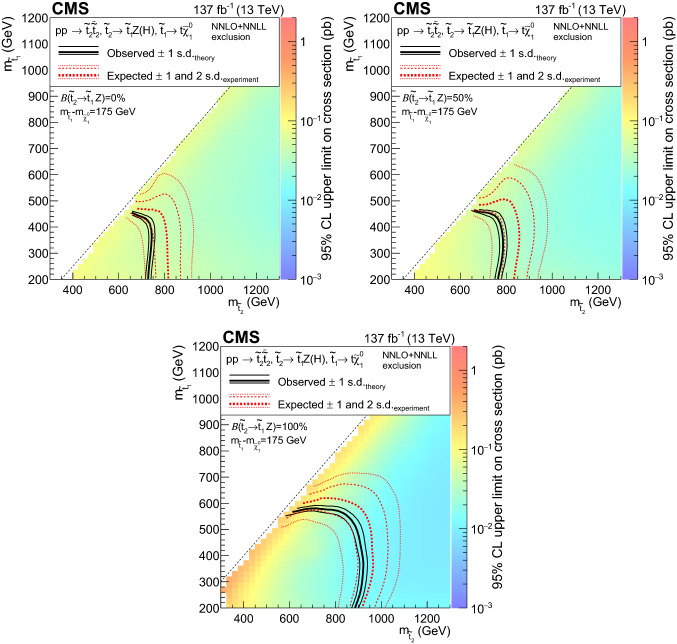
Exclusion regions at 95% ${\text {CL}}$ in the plane of $m({\tilde{\text {t}}} _{1})$ versus $m({\tilde{\text {t}}} _{2})$ for the $\mathrm {T}6{{\text {ttHZ}}}$ model with $m({\tilde{\text {t}}} _{1})-m({\tilde{\chi }}^{0}_{1})=175\,{\text {GeV}}$. The three exclusions represent ${\mathcal {B}}({\tilde{\text {t}}} _{2}\rightarrow {\tilde{\text {t}}} _{1}{\text {Z}})$ of 0, 50, and 100%, respectively. The notations are as in Fig. 7

Figure 7 shows observed and expected exclusion limits for simplified models of gluino pair production with each gluino decaying to off- or on-shell third-generation squarks. These models were introduced in Sect. 2 and denoted as $\mathrm {T}1{{\text {tttt}}}$, $\mathrm {T}5{{\text {ttbbWW}}}$, $\mathrm {T}5{{\text {tttt}}}$, and $\mathrm {T}5{{\text {ttcc}}}$. Similarly, Figs. 8 and 9 show the corresponding limits for $\mathrm {T}5{{\text {qqqqWZ}}}$ and $\mathrm {T}5{{\text {qqqqWW}}}$, with two different assumptions on the chargino mass. Note that the $\mathrm {T}5{{\text {qqqqWZ}}}$ model assumes equal probabilities for the decay of the gluino into ${\tilde{\chi }}^{+}_{1}$, ${\tilde{\chi }}^{-}_{1}$, and ${\tilde{\chi }} _{2}^{0}$. The exclusion limits for $\mathrm {T}6{{\text {ttWW}}}$ and $\mathrm {T}6{{\text {ttHZ}}}$ are displayed in Figs. 10 and 11, respectively. In the $\mathrm {T}6{{\text {ttHZ}}}$ model, the heavier top squark decays into a lighter top squark and a ${\text {Z}}$ or ${\text {H}}$ boson. The three sets of exclusion limits shown in Fig. 11 correspond to the branching fraction ${\mathcal {B}}({\tilde{\text {t}}} _{2}\rightarrow {\tilde{\text {t}}} _{1}{\text {Z}})$ having values of 0, 50, and 100%.

**Fig. 12:**
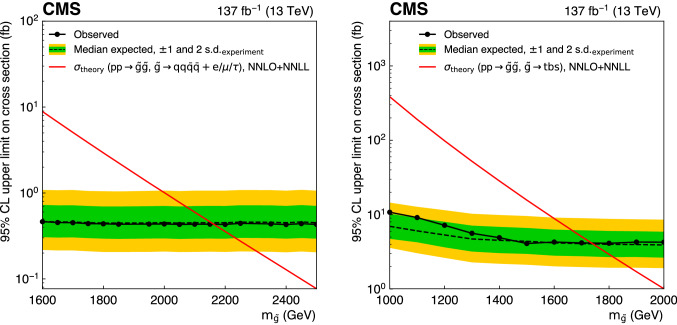
Upper limits at 95% ${\text {CL}}$ on the cross section for RPV gluino pair production with each gluino decaying into four quarks and one lepton ($\mathrm {T}1{{\text {qqqqL}}}$, left), and each gluino decaying into a top, bottom, and strange quarks ($\mathrm {T}1{{\text {tbs}}}$, right)

Finally, Fig. 12 shows observed and expected limits on the cross section of gluino pair production as a function of the gluino masses in the two RPV models described in Sect. 2. The observed and expected exclusions on the gluino mass are similar and reach 2.1 and $1.7\,{\text {TeV}}$ for the $\mathrm {T}1{{\text {qqqqL}}}$ and $\mathrm {T}1{{\text {tbs}}}$ models, respectively.

The analysis sensitivity for the various models studied in Figs. 7, 8, 9, 10 and 11 is often driven by the event yields in a few SRs (off-$\text {Z}$ ML21, HH53 and HH52), where a slight excess of data is observed. This in particular applies to the uncompressed mass regime, resulting in an observed limit weaker than the expected one by one or two s.d. In the compressed mass regime, however, other SRs can become dominant, for example when the hadronic activity becomes limited. This happens in the $\mathrm {T}5{{\text {qqqqWZ}}}$ and $\mathrm {T}5{{\text {qqqqWW}}}$ models where the gluino and the lightest neutralino present a limited mass splitting (the region close to the diagonal in the left plots of Figs. 8, 9). In those scenarios the on-${\text {Z}}$ ML4 and HH3 SRs provide the best sensitivity, respectively. Additionally, if the intermediate chargino is nearly degenerate in mass with the lightest neutralino, both leptons become soft and LL SRs such as LL2 become relevant. Such a situation is encountered in the phase space region close to the diagonal in the right plots of Figs. 8 and  9. On-${\text {Z}}$ SRs (especially on-${\text {Z}}$ ML23) become important for models where an on-shell ${\text {Z}}$ boson is produced (bottom plot in Fig. 11). The limits on the RPV models presented in Fig. 12 are mostly driven by another set of SRs (HH62 and LM11, the latter becoming more relevant for lower masses).

Compared to the previous versions of the analysis [24, 25], the limits for the RPC models extend the gluino and squark mass observed and expected exclusions by up to $200\,{\text {GeV}}$ because of the increase in the integrated luminosity and the corresponding re-optimization of SR definitions. These results also complement searches for gluino pair production conducted by CMS in final states with 0 or 1 lepton [86–88]. For the $\mathrm {T}1{{\text {tttt}}}$ scenario, the expected sensitivity of this analysis suffers from a lower branching fraction that makes it uncompetitive in the uncompressed mass regime. However, for a nearly degenerate mass spectrum, the SM background becomes of higher importance and the presence of an SS lepton pair significantly reduces it, leading to a similar sensitivity. The constraints on the two RPV models that were not previously included demonstrate the sensitivity of the analysis to RPV scenarios. The final state is particularly well suited to study the $\mathrm {T}1{{\text {qqqqL}}}$ model since no leptonic branching fraction penalty applies, resulting in exclusion limits on the gluino mass beyond 2.1 TeV, comparable to other results in fully hadronic final states [87, 88]. The limits obtained on the $\mathrm {T}1{{\text {tbs}}}$ model are stronger than those previously obtained in the one-lepton channel based on the analysis of the 2016 dataset [89]. They are expected to remain competitive after an update with the full Run 2 dataset.

**Fig. 13:**
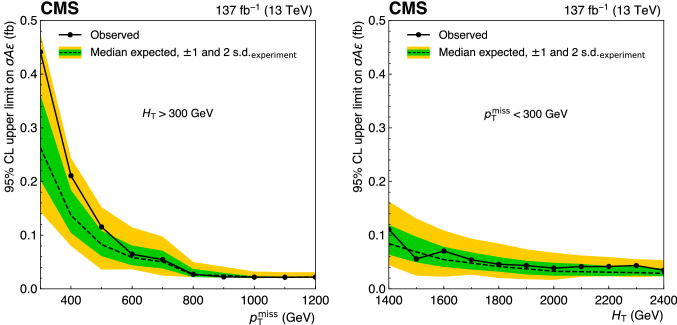
Upper limits at 95% ${\text {CL}}$ on the product of cross section, detector acceptance, and selection efficiency, $\sigma \! {\mathcal {A}} \epsilon$, for the production of an SS lepton pair with at least two jets, as a function of the minimum $p_{{\mathrm {T}}} ^{\text {miss}}$ threshold, when $H_{\text {T}} >300\,{\text {GeV}}$ (left), or the minimum $H_{\text {T}}$ threshold, when $p_{{\mathrm {T}}} ^{\text {miss}} <300\,{\text {GeV}}$ (right)

Model-independent limits are also set on the product of cross section, branching fraction, detector acceptance, and reconstruction efficiency, for the production of an SS lepton pair with at least two extra jets and $H_{\text {T}} >300\,{\text {GeV}}$. For this purpose, we select events from the HH and LM categories and calculate limits as a function of minimum $p_{{\mathrm {T}}} ^{\text {miss}}$ or $H_{\text {T}}$ requirements starting at 300 and $1400\,{\text {GeV}}$, respectively. In order to remove the overlap between the two conditions, events selected for the $H_{\text {T}}$ scan must also satisfy $p_{{\mathrm {T}}} ^{\text {miss}} <300\,{\text {GeV}}$. The corresponding limits are presented in Fig. 13.

Finally, in order to facilitate reinterpretations of our results, we present in Table 10 the expected and observed yields for a number of inclusive SRs. This procedure focuses on events with large $H_{\text {T}}$, $p_{{\mathrm {T}}} ^{\text {miss}}$, $N_{{\text {b}}}$, and/or $N_\text {jets}$, and the SRs are defined such that they typically lead to 5 to 10 expected background events. The last column in the table indicates the upper limit at 95% ${\text {CL}}$ on the number of BSM events in each SR.

**Table 10:** Inclusive SR definitions, expected background yields and uncertainties, and observed yields, as well as the observed 95% ${\text {CL}}$ upper limits on the number of BSM events contributing to each region. No uncertainty in the signal acceptance is assumed in calculating these limits. A dash (—) indicates that a particular selection is not required

SR	Category	$N_\text {jets}$	$N_{{\text {b}}}$	$H_{\text {T}}$ (${\text {GeV}}$)	$p_{{\mathrm {T}}} ^{\text {miss}}$ (${\text {GeV}}$)	$m_{\text {T}} ^{\text {min}}$ (${\text {GeV}}$)	SM expected	Obs.	$N_{{\text {BSM}}}^{{\text {max}}}(95\%\, {\text {CL}})$
ISR1	HH	$\ge$2	0	$\ge$1000	$\ge$250	—	$12.7 \pm 7.4$	16	12.32
ISR2		$\ge$2	$\ge$2	$\ge$1100	—	—	$11.0 \pm 3.8$	14	11.33
ISR3		$\ge$2	0	—	$\ge$500	—	$10.4 \pm 9.7$	13	11.26
ISR4		$\ge$2	$\ge$2	—	$\ge$300	—	$11.4 \pm 3.8$	17	14.22
ISR5		$\ge$2	0	—	$\ge$250	$\ge$120	$6.6 \pm 5.7$	10	10.77
ISR6		$\ge$2	$\ge$2	—	$\ge$200	$\ge$120	$6.3 \pm 1.3$	8	8.22
ISR7		$\ge$8	—	—	—	—	$7.0 \pm 2.8$	12	12.17
ISR8		$\ge$6	—	—	—	$\ge$120	$6.2 \pm 1.4$	10	10.45
ISR9		$\ge$2	$\ge$3	$\ge$800	—	—	$7.8 \pm 3.5$	8	7.53
ISR10	LL	$\ge$2	—	$\ge$700	—	—	$10.4 \pm 9.0$	12	10.37
ISR11		$\ge$2	—	—	$\ge$200	—	$12.1 \pm 5.6$	13	9.94
ISR12		$\ge$6	—	—	—	—	$7.1 \pm 4.3$	7	7.10
ISR13		$\ge$2	$\ge$3	—	—	—	$1.61 \pm 0.39$	3	5.70
ISR14	LM	$\ge$2	0	$\ge$1200	${<}50$	—	$3.6 \pm 3.6$	3	5.10
ISR15		$\ge$2	$\ge$2	$\ge$1000	${<}50$	—	$2.34 \pm 0.51$	4	6.41
ISR16	ML	$\ge$2	0	$\ge$1000	$\ge$300	—	$5.6 \pm 1.6$	7	7.78
ISR17		$\ge$2	$\ge$2	$\ge$1000	—	—	$5.7 \pm 1.9$	7	7.62

## Summary

A sample of events with two same-sign or at least three charged leptons (electrons or muons) produced in association with several jets in proton-proton collisions at $13\,{\text {TeV}}$, corresponding to an integrated luminosity of $137{\,{\text {fb}}^{-1}}$, has been studied to search for manifestations of physics beyond the standard model. The data are found to be consistent with the standard model expectations. The results are interpreted as limits on cross sections at 95% confidence level for the production of new particles in simplified supersymmetric models, considering both R parity conserving and violating scenarios. Using calculations for these cross sections as functions of particle masses, the limits are translated into lower mass limits that are as large as $2.1\,{\text {TeV}}$ for gluinos and $0.9\,{\text {TeV}}$ for top and bottom squarks, depending on the details of the model. The results extend the gluino and squark mass observed and expected exclusions by up to $200\,{\text {GeV}}$, compared to the previous versions of this analysis. Finally, to facilitate further interpretations of the search, model-independent limits are provided as a function of the missing transverse momentum and the scalar sum of jet transverse momenta in an event, together with the background prediction and data yields in a set of simplified signal regions.

## Data Availability

This manuscript has no associated data or the data will not be deposited. [Authors' comment: Release and preservation of data used by the CMS Collaboration as the basis for publications is guided by the CMS policy as written in its document "CMS data preservation, re-use and open access policy" (https://cms-docdb.cern.ch/cgi-bin/PublicDocDB/RetrieveFile?docid=6032&filename=CMSDataPolicyV1.2.pdf&version=2.]

## Appendix A: Extended results

Tables 11, 12, 13, 14, 15 and 16, corresponding to Figs. 5 and 6, show background predictions per process within each signal region.

**Table 11:** Event yields in HH regions. Yields shown as “–” have a contribution smaller than 0.01, or do not contribute to a particular region

	$\hbox {t}{\bar{\hbox {t}}} {\text {W}}$	$\hbox {t}{\bar{\hbox {t}}} {\text {Z}}$	$\hbox {t}{\bar{\hbox {t}}} {\text {H}}$	${\text {W}}$ ${\text {Z}}$	WW	X+$\gamma$	Rare	Charge misid.	Nonprompt lep.	SM expected	Data
SR1	53±15	14.1±4.1	13.7±3.5	349±97	129±37	300±120	105±43	136±16	460±260	1560±300	1673
SR2	28.5±8.1	7.7±2.2	7.7±2.0	124±34	161±46	86±40	38±16	34.6±3.9	94±60	582±93	653
SR3	8.1±2.3	1.46±0.41	1.64±0.42	19.8±5.6	13.0±3.8	7.3±7.3	8.8±3.7	14.0±1.6	26±21	100±25	128
SR4	6.2±1.8	2.34±0.67	3.45±0.86	4.0±1.1	4.4±1.3	0.50±0.23	4.3±1.8	2.01±0.22	12.4±7.7	39.5±8.5	54
SR5	4.0±1.1	0.48±0.14	0.49±0.12	15.9±4.4	21.1±6.1	5.5±4.7	4.8±2.0	0.50±0.06	4.9±2.4	57.7±9.9	53
SR6	1.64±0.49	0.49±0.14	0.57±0.15	6.7±2.0	5.2±1.6	8.6±5.3	2.6±1.0	0.61±0.07	6.0±3.3	32.5±7.1	24
SR7	0.91±0.26	0.26±0.07	0.38±0.09	0.62±0.19	0.84±0.23	1.2±1.2	0.43±0.18	0.08±0.01	0.81±0.51	5.5±1.8	7
SR8	1.67±0.48	0.27±0.09	0.24±0.06	3.10±0.89	6.9±2.0	2.6±2.6	1.83±0.81	4.15±0.48	2.1±2.0	22.9±5.1	33
SR9	1.00±0.29	0.27±0.09	0.20±0.05	2.79±0.77	4.5±1.3	2.0±2.0	1.17±0.50	4.59±0.54	3.0±2.4	19.5±3.9	20
SR10	1.45±0.42	0.26±0.07	0.27±0.07	0.99±0.27	2.71±0.82	0.02±0.02	1.66±0.68	0.66±0.08	1.61±0.84	9.6±1.9	11
SR11	130±37	34.6±9.8	35.0±9.2	29.8±8.3	11.3±3.3	49±21	30±12	89±10	530±270	940±270	1115
SR12	80±22	21.2±6.0	22.3±5.7	14.2±3.9	15.8±4.7	24±11	16.3±6.7	21.7±2.5	125±76	340±81	384
SR13	12.8±3.6	1.96±0.56	1.97±0.51	1.54±0.44	0.77±0.29	1.43±0.90	1.35±0.54	3.91±0.44	10.6±8.3	36.3±9.5	40
SR14	6.5±1.8	1.93±0.56	1.96±0.51	0.54±0.19	0.31±0.13	1.05±0.57	1.03±0.42	4.15±0.47	9.4±6.9	26.8±7.4	26
SR15	13.9±3.9	4.0±1.2	6.4±1.6	0.44±0.15	0.67±0.20	0.67±0.32	3.5±1.4	1.16±0.13	12.1±7.0	42.7±8.6	68
SR16	7.8±2.2	4.0±1.2	6.2±1.6	0.35±0.11	0.35±0.10	0.86±0.41	2.7±1.1	1.18±0.13	14.4±7.9	37.9±8.6	41
SR17	9.3±2.7	1.21±0.35	1.38±0.35	1.86±0.53	1.96±0.57	3.3±3.3	1.74±0.70	0.89±0.10	4.9±3.1	26.5±6.2	29
SR18	4.1±1.2	1.05±0.31	1.30±0.33	0.72±0.21	0.54±0.16	0.76±0.39	0.94±0.39	0.95±0.11	4.0±3.1	14.3±3.6	13
SR19	2.76±0.78	0.87±0.25	1.24±0.32	0.14±0.04	0.14±0.04	1.6±1.5	0.73±0.31	0.20±0.02	2.9±1.5	10.6±2.5	12
SR20	4.9±1.4	0.76±0.22	0.60±0.15	0.54±0.17	0.66±0.19	1.7±1.7	0.72±0.31	1.17±0.13	1.2±1.2	12.3±2.9	14
SR21	2.66±0.74	0.68±0.20	0.59±0.15	0.27±0.13	0.62±0.18	0.53±0.30	0.59±0.25	1.27±0.14	1.9±1.9	9.2±2.7	17
SR22	4.5±1.3	0.75±0.22	1.00±0.25	0.16±0.06	0.30±0.09	0.42±0.19	1.30±0.55	0.49±0.05	1.2±1.2	10.1±2.1	17
SR23	77±22	20.5±5.9	22.2±6.0	1.62±0.51	0.59±0.17	22.4±9.3	8.8±3.7	56.9±6.5	61±31	272±43	354
SR24	55±16	14.8±4.2	16.8±4.4	1.16±0.35	0.85±0.25	9.4±3.6	7.8±3.2	13.6±1.6	27±15	147±25	177
SR25	7.5±2.2	1.05±0.30	1.19±0.32	0.09±0.03	–	0.60±0.35	0.79±0.33	2.18±0.25	1.9±1.7	15.3±2.9	12
SR26	4.1±1.2	0.89±0.28	1.29±0.34	0.02±0.02	0.02±0.01	0.76±0.35	0.53±0.22	2.14±0.25	1.7±1.7	11.4±2.4	19
SR27	12.2±3.6	3.7±1.1	6.0±1.6	0.15±0.06	0.05±0.03	1.01±0.49	3.9±1.6	0.96±0.11	5.4±2.8	33.4±5.4	49
SR28	7.4±2.1	3.6±1.1	6.0±1.6	0.02±0.01	0.12±0.04	0.95±0.40	3.3±1.4	1.06±0.12	7.6±3.3	30.1±4.9	38
SR29	5.4±1.5	0.61±0.18	0.79±0.21	0.16±0.06	0.04±0.03	0.16±0.09	0.65±0.27	0.39±0.04	2.2±1.5	10.4±2.2	9
SR30	2.68±0.80	0.57±0.15	0.80±0.21	0.20±0.06	0.08±0.03	0.47±0.23	0.37±0.15	0.48±0.06	0.94±0.79	6.6±1.3	7
SR31	2.74±0.84	0.56±0.17	1.16±0.30	0.06±0.02	0.07±0.03	0.15±0.06	1.10±0.46	0.18±0.02	0.92±0.92	6.9±1.5	6
SR32	3.41±0.96	0.60±0.18	0.47±0.12	–	0.05±0.02	0.35±0.17	0.38±0.16	0.52±0.06	0.10±0.10	5.9±1.1	14
SR33	2.09±0.60	0.59±0.18	0.58±0.15	–	0.01±0.01	0.24±0.12	0.31±0.13	0.55±0.06	1.7±1.3	6.1±1.6	7
SR34	2.97±0.90	0.67±0.20	0.81±0.21	0.02±0.00	0.08±0.03	0.24±0.10	1.12±0.46	0.33±0.04	0.58±0.58	6.8±1.3	10
SR35	3.25±0.95	0.83±0.26	1.19±0.33	–	–	0.27±0.22	0.57±0.25	0.95±0.11	1.75±0.98	8.8±1.5	16
SR36	1.83±0.55	0.80±0.24	1.22±0.34	–	–	0.48±0.28	0.49±0.21	0.97±0.11	2.9±1.8	8.7±2.0	11
SR37	3.3±1.0	0.98±0.30	1.11±0.31	–	0.02±0.01	0.40±0.17	1.03±0.42	0.55±0.06	2.0±1.3	9.4±1.9	7
SR38	1.93±0.58	0.89±0.26	1.07±0.30	0.01±0.01	–	0.38±0.27	0.97±0.40	0.56±0.07	1.17±0.80	7.0±1.3	5
SR39	2.16±0.65	0.65±0.21	1.16±0.33	0.02±0.01	–	0.22±0.09	3.4±1.4	0.20±0.02	1.7±1.1	9.6±2.1	9
SR40	1.54±0.49	0.82±0.28	1.29±0.36	–	–	0.36±0.17	3.1±1.3	0.21±0.03	1.26±0.67	8.6±1.7	11
SR41	0.46±0.14	0.14±0.04	0.16±0.05	–	–	0.07±0.04	0.13±0.05	0.14±0.02	–	1.10±0.32	2
SR42	0.24±0.09	0.08±0.05	0.08±0.03	–	–	0.02±0.02	0.17±0.07	0.04±0.01	–	0.63±0.49	0
SR43	0.21±0.09	0.04±0.04	0.07±0.02	–	–	0.12±0.12	0.14±0.06	0.04±0.01	0.04±0.04	0.67±0.60	1
SR44	0.14±0.04	0.03±0.01	0.08±0.02	–	–	0.02±0.01	0.39±0.16	0.04±0.00	0.04±0.04	0.74±0.27	1
SR45	0.16±0.08	0.04±0.04	0.07±0.03	0.03±0.03	–	0.04±0.04	0.36±0.15	0.01±0.01	–	0.71±0.53	1
SR46	9.6±2.8	0.93±0.26	0.91±0.23	8.3±2.3	14.7±4.3	6.9±6.8	3.5±1.5	0.61±0.07	2.3±2.0	47.8±9.7	59
SR47	3.5±1.0	0.84±0.28	0.92±0.23	3.04±0.84	2.92±0.88	1.9±1.9	1.51±0.62	0.66±0.07	2.0±1.4	17.3±3.8	24
SR48	1.27±0.40	0.12±0.04	0.05±0.02	1.64±0.46	4.7±1.4	1.8±1.8	0.44±0.18	0.05±0.01	0.20±0.16	10.3±2.9	11
SR49	0.37±0.11	0.13±0.04	0.08±0.02	0.62±0.23	0.41±0.13	0.02±0.01	0.34±0.17	0.04±0.00	0.03±0.03	2.06±0.49	3
SR50	2.57±0.79	0.30±0.10	0.54±0.15	0.43±0.12	0.72±0.23	0.06±0.03	1.11±0.45	0.09±0.01	0.72±0.31	6.5±1.1	13
SR51	1.16±0.38	0.35±0.14	0.57±0.15	0.20±0.07	0.15±0.06	0.02±0.01	0.79±0.34	0.08±0.01	0.40±0.38	3.72±0.79	4
SR52	0.45±0.12	0.06±0.02	0.05±0.02	0.22±0.09	0.15±0.05	0.02±0.01	0.20±0.08	–	0.05±0.05	1.21±0.29	4
SR53	0.20±0.09	0.05±0.04	0.06±0.03	–	0.03±0.03	–	0.08±0.04	0.01±0.01	–	0.44±0.44	2
SR54	1.75±0.53	0.26±0.09	0.23±0.07	0.95±0.29	4.6±1.4	0.65±0.58	0.63±0.27	0.46±0.05	0.29±0.12	9.8±1.8	24
SR55	0.99±0.28	0.15±0.05	0.12±0.04	0.78±0.23	4.0±1.1	0.03±0.03	0.58±0.24	0.35±0.04	0.33±0.33	7.3±1.4	4
SR56	0.57±0.16	0.04±0.01	0.07±0.02	0.39±0.13	2.46±0.72	0.34±0.34	0.17±0.07	0.18±0.02	0.22±0.17	4.44±0.98	6
SR57	2.04±0.59	0.48±0.13	0.53±0.16	0.29±0.10	0.47±0.15	0.04±0.02	0.71±0.29	0.22±0.03	0.89±0.73	5.7±1.1	6
SR58	1.65±0.56	0.17±0.05	0.30±0.09	0.14±0.05	0.47±0.15	–	0.70±0.31	0.13±0.02	0.48±0.48	4.0±1.0	6
SR59	0.99±0.35	0.06±0.02	0.16±0.06	0.15±0.06	0.37±0.11	–	0.27±0.12	0.13±0.01	0.11±0.11	2.24±0.53	2
SR60	0.61±0.18	0.08±0.04	0.33±0.09	–	0.01±0.01	0.03±0.02	0.67±0.29	0.04±0.00	0.06±0.06	1.83±0.44	5
SR61	0.65±0.21	0.12±0.04	0.20±0.06	0.08±0.05	0.09±0.03	–	0.50±0.20	0.03±0.00	0.19±0.14	1.88±0.40	5
SR62	0.41±0.12	0.05±0.02	0.14±0.05	0.04±0.01	0.03±0.01	0.01±0.01	0.22±0.09	0.02±0.00	0.43±0.43	1.35±0.56	0
Total	610±180	158±40	180±44	600±160	420±120	540±250	280±150	409±43	1460±740	4660±880	5376

**Table 12:** Event yields in HL regions. Yields shown as “–” have a contribution smaller than 0.01, or do not contribute to a particular region

	$\hbox {t}{\bar{\hbox {t}}} {\text {W}}$	$\hbox {t}{\bar{\hbox {t}}} {\text {Z}}$	$\hbox {t}{\bar{\hbox {t}}} {\text {H}}$	${\text {W}}$ ${\text {Z}}$	WW	X+$\gamma$	Rare	Charge misid.	Nonprompt lep.	SM expected	Data
SR1	17.5±4.9	6.2±1.8	6.8±1.8	168±47	49±14	145±67	51±21	33.6±3.8	910±290	1390±300	1593
SR2	7.2±2.1	2.63±0.76	3.21±0.82	38±10	46±13	41±25	11.3±4.8	6.54±0.73	192±62	348±67	337
SR3	0.97±0.28	0.20±0.07	0.34±0.09	5.0±1.4	2.24±0.68	0.04±0.04	1.88±0.79	0.24±0.03	16.0±5.3	26.9±8.8	39
SR4	1.85±0.54	0.81±0.23	1.67±0.42	1.19±0.34	1.45±0.42	0.30±0.13	1.25±0.50	0.48±0.06	26.9±9.1	35.9±9.1	34
SR5	1.15±0.33	0.20±0.06	0.23±0.06	5.5±1.5	7.8±2.3	4.2±3.7	1.12±0.46	0.10±0.01	9.4±3.0	29.8±6.0	34
SR6	0.36±0.10	0.21±0.07	0.22±0.06	2.73±0.76	1.43±0.43	8.4±6.7	0.49±0.22	0.19±0.02	8.2±2.5	22.2±7.2	12
SR7	0.22±0.07	0.10±0.05	0.19±0.05	0.29±0.10	0.25±0.09	0.07±0.04	0.32±0.14	0.02±0.00	3.2±1.4	4.7±1.4	6
SR8	46±13	16.5±4.7	18.0±4.7	14.6±4.0	3.4±1.0	36±16	13.5±5.5	25.4±2.9	920±280	1100±280	1342
SR9	22.0±6.2	8.2±2.3	9.9±2.5	4.4±1.2	4.1±1.2	8.8±3.8	4.6±1.9	4.53±0.51	232±70	299±71	330
SR10	1.65±0.48	0.27±0.08	0.35±0.09	0.16±0.06	0.16±0.05	0.64±0.29	0.22±0.11	0.23±0.03	5.5±2.1	9.1±2.3	8
SR11	0.81±0.25	0.23±0.08	0.41±0.11	0.12±0.05	0.05±0.02	0.17±0.09	0.21±0.09	0.20±0.02	4.2±1.6	6.4±1.6	9
SR12	4.2±1.2	1.26±0.38	3.23±0.82	0.08±0.03	0.25±0.10	1.58±0.86	0.85±0.34	0.29±0.03	30.3±9.0	42.1±9.2	49
SR13	1.95±0.58	1.37±0.41	3.18±0.81	0.10±0.06	0.06±0.02	1.14±0.44	0.92±0.38	0.32±0.04	24.0±8.2	33.0±8.4	39
SR14	4.3±1.3	0.88±0.25	1.16±0.30	1.14±0.33	0.90±0.26	0.51±0.23	0.59±0.25	0.45±0.05	15.9±5.5	25.8±5.9	25
SR15	0.62±0.23	0.10±0.10	0.30±0.09	–	0.04±0.04	0.03±0.03	0.15±0.08	0.03±0.01	1.6±1.6	2.8±2.0	7
SR16	0.30±0.11	0.17±0.08	0.34±0.09	0.06±0.06	0.02±0.02	–	0.14±0.07	0.03±0.01	1.4±1.2	2.5±1.3	2
SR17	28.9±8.2	10.3±3.0	11.7±3.1	0.68±0.21	0.09±0.04	15.6±6.7	3.9±1.6	17.9±2.1	133±39	222±42	260
SR18	16.8±4.8	6.2±1.8	7.9±2.1	0.53±0.15	0.24±0.08	6.4±2.6	2.40±1.00	3.34±0.38	43±13	86±15	104
SR19	0.72±0.24	0.12±0.04	0.28±0.07	–	–	0.04±0.04	0.05±0.03	0.13±0.01	0.87±0.69	2.22±0.90	4
SR20	0.58±0.17	0.11±0.04	0.26±0.07	0.05±0.03	0.03±0.02	0.12±0.05	0.09±0.04	0.13±0.01	1.8±1.1	3.2±1.1	4
SR21	4.4±1.3	1.69±0.47	3.13±0.82	–	0.11±0.03	2.5±1.9	1.23±0.50	0.25±0.03	6.5±2.2	19.8±3.8	28
SR22	2.06±0.57	1.41±0.41	3.15±0.82	–	–	1.01±0.49	0.95±0.38	0.23±0.03	7.3±2.5	16.1±3.0	19
SR23	1.96±0.56	0.21±0.08	0.42±0.11	0.05±0.02	0.09±0.04	0.21±0.09	0.16±0.07	0.12±0.01	1.5±1.0	4.7±1.3	1
SR24	0.83±0.25	0.27±0.09	0.37±0.10	0.09±0.04	0.01±0.01	0.17±0.08	0.16±0.06	0.15±0.02	1.9±1.2	4.0±1.2	2
SR25	1.11±0.34	0.26±0.08	0.57±0.15	–	–	0.21±0.09	0.31±0.13	0.05±0.01	1.52±0.98	4.0±1.1	5
SR26	1.02±0.30	0.41±0.12	0.61±0.17	–	0.01±0.00	0.49±0.20	0.21±0.09	0.27±0.03	5.4±2.3	8.5±2.4	7
SR27	0.53±0.15	0.47±0.14	0.60±0.17	–	–	0.53±0.22	0.17±0.08	0.28±0.03	5.8±2.5	8.4±2.5	7
SR28	1.36±0.41	0.60±0.20	1.14±0.31	–	–	0.21±0.09	1.02±0.42	0.18±0.02	4.4±2.0	8.9±2.2	11
SR29	0.92±0.28	0.43±0.14	1.12±0.31	–	–	0.34±0.14	0.98±0.41	0.22±0.03	6.9±3.0	10.9±3.1	11
SR30	0.36±0.13	0.15±0.05	0.20±0.06	–	–	0.02±0.01	0.30±0.12	0.03±0.00	0.18±0.18	1.25±0.39	3
SR31	0.41±0.12	0.05±0.02	0.17±0.04	0.15±0.05	0.12±0.05	0.05±0.02	0.08±0.04	0.09±0.01	0.81±0.29	1.92±0.37	4
SR32	0.73±0.24	0.06±0.02	0.19±0.05	0.18±0.07	0.31±0.11	0.06±0.03	0.33±0.14	0.07±0.01	0.83±0.38	2.77±0.56	3
SR33	2.51±0.71	0.32±0.08	0.33±0.09	2.66±0.76	5.0±1.4	0.16±0.16	1.62±0.69	0.12±0.01	6.4±1.9	19.1±4.1	23
SR34	0.99±0.27	0.24±0.07	0.30±0.08	1.19±0.34	0.70±0.21	0.08±0.05	0.72±0.32	0.11±0.01	3.2±1.2	7.5±1.5	9
SR35	0.30±0.09	0.03±0.01	0.03±0.01	0.55±0.17	0.95±0.30	0.01±0.01	0.15±0.08	–	0.09±0.09	2.12±0.49	5
SR36	0.11±0.05	0.02±0.02	–	0.10±0.10	0.06±0.06	–	0.08±0.08	–	0.09±0.09	0.47±0.33	1
SR37	0.71±0.21	0.13±0.04	0.20±0.05	0.18±0.06	0.34±0.10	0.01±0.01	0.19±0.09	0.02±0.00	0.96±0.65	2.75±0.77	4
SR38	0.19±0.06	0.11±0.04	0.21±0.06	0.02±0.02	0.06±0.02	0.01±0.01	0.18±0.08	0.02±0.00	0.88±0.45	1.68±0.50	0
SR39	0.21±0.08	0.02±0.02	0.03±0.01	–	0.07±0.06	–	0.04±0.02	–	0.59±0.59	0.97±0.97	0
SR40	0.60±0.17	0.05±0.04	0.18±0.05	0.11±0.08	1.01±0.30	–	0.30±0.13	0.05±0.01	0.54±0.43	2.83±0.70	7
SR41	0.24±0.12	0.12±0.07	0.18±0.06	0.05±0.05	0.17±0.12	2.1±2.1	0.11±0.06	0.04±0.01	0.79±0.54	3.8±3.8	0
SR42	0.92±0.29	0.12±0.04	0.15±0.05	0.27±0.08	1.59±0.46	0.03±0.01	0.21±0.09	0.06±0.01	1.57±0.81	4.9±1.0	9
SR43	0.24±0.07	0.11±0.04	0.21±0.07	0.16±0.05	0.32±0.10	0.51±0.51	0.19±0.08	0.09±0.01	0.53±0.20	2.36±0.72	5
Total	180±52	63±16	83±20	248±64	129±36	270±120	105±56	97±10	2640±590	3820±620	4402

**Table 13:** Event yields in LL regions. Yields shown as “–” have a contribution smaller than 0.01, or do not contribute to a particular region

	$\hbox {t}{\bar{\hbox {t}}} {\text {W}}$	$\hbox {t}{\bar{\hbox {t}}} {\text {Z}}$	$\hbox {t}{\bar{\hbox {t}}} {\text {H}}$	${\text {W}}$ ${\text {Z}}$	WW	X+$\gamma$	Rare	Charge misid.	Nonprompt lep.	SM expected	Data
SR1	0.34±0.10	0.27±0.08	0.30±0.08	1.93±0.57	2.27±0.66	0.41±0.28	0.38±0.17	0.06±0.01	17.0±7.2	23.0±7.2	29
SR2	0.10±0.04	0.05±0.03	0.07±0.02	0.93±0.31	0.86±0.25	0.03±0.01	0.37±0.16	–	2.6±1.5	5.0±1.6	6
SR3	1.00±0.27	0.90±0.26	1.03±0.26	0.19±0.06	0.14±0.04	0.14±0.14	0.33±0.14	0.09±0.01	19.9±6.5	23.8±6.6	27
SR4	0.47±0.14	0.11±0.03	0.28±0.07	0.05±0.02	0.08±0.02	0.09±0.04	0.19±0.08	0.01±0.00	3.4±1.5	4.7±1.5	7
SR5	0.92±0.26	0.68±0.20	0.96±0.25	0.01±0.00	0.02±0.02	0.16±0.14	0.28±0.12	0.09±0.01	4.8±1.8	8.0±1.9	15
SR6	0.33±0.13	0.14±0.07	0.21±0.06	–	0.04±0.04	0.03±0.03	0.05±0.02	0.02±0.01	1.2±1.1	2.0±1.1	0
SR7	0.17±0.06	0.09±0.03	0.19±0.06	–	–	0.12±0.05	0.21±0.09	0.02±0.00	0.81±0.56	1.61±0.59	3
SR8	0.02±0.02	0.01±0.01	0.01±0.01	–	–	–	–	–	–	0.06±0.06	0
Total	3.36±0.98	2.25±0.60	3.04±0.80	3.09±0.82	3.42±0.96	0.79±0.42	1.84±0.97	0.30±0.03	50±13	68±13	87

**Table 14:** Event yields in on-${\text {Z}}$ ML regions. Yields shown as “–” have a contribution smaller than 0.01, or do not contribute to a particular region

	$\hbox {t}{\bar{\hbox {t}}} {\text {W}}$	$\hbox {t}{\bar{\hbox {t}}} {\text {Z}}$	$\hbox {t}{\bar{\hbox {t}}} {\text {H}}$	${\text {W}}$ ${\text {Z}}$	X+$\gamma$	Rare	Nonprompt lep.	SM expected	Data
SR1	1.12±0.30	46±13	1.67±0.43	620±170	11.9±5.7	105±42	54±18	840±170	985
SR2	0.71±0.21	6.8±1.9	0.46±0.12	68±19	3.1±2.6	17.8±7.3	9.9±4.6	107±21	136
SR3	0.18±0.05	8.5±2.4	0.23±0.06	95±26	0.02±0.01	11.7±4.8	3.2±1.8	119±27	146
SR4	0.17±0.06	1.99±0.54	0.17±0.04	5.8±1.6	0.03±0.01	2.19±0.87	0.68±0.46	11.1±2.1	10
SR5	0.13±0.04	7.7±2.2	0.25±0.06	83±23	2.3±1.2	11.0±4.5	4.5±2.1	109±24	126
SR6	0.09±0.03	1.42±0.39	0.06±0.02	13.6±3.8	0.74±0.61	2.5±1.0	0.93±0.69	19.3±4.1	24
SR7	0.10±0.03	2.55±0.73	0.09±0.02	35±10	0.09±0.04	3.7±1.6	0.89±0.53	42±10	47
SR8	0.11±0.03	0.45±0.15	0.04±0.01	2.02±0.67	–	0.61±0.25	0.25±0.18	3.47±0.84	3
SR9	5.6±1.6	140±39	6.1±1.6	52±14	5.3±2.4	72±29	46±17	327±54	419
SR10	1.70±0.48	23.4±6.7	1.18±0.31	7.6±2.1	0.20±0.09	9.1±3.8	3.2±2.0	46.5±8.4	53
SR11	0.61±0.17	25.9±7.5	0.98±0.25	11.0±3.0	0.27±0.16	9.5±3.9	3.1±1.3	51.3±9.1	62
SR12	0.30±0.08	7.5±2.1	0.36±0.09	3.48±0.99	0.04±0.02	2.9±1.2	0.95±0.67	15.6±2.8	27
SR13	4.8±1.4	89±25	4.5±1.2	3.7±1.0	2.17±0.90	22.4±9.0	4.9±1.9	131±27	162
SR14	0.98±0.29	13.4±3.9	0.76±0.20	0.62±0.19	0.09±0.03	2.8±1.1	1.24±0.83	19.9±4.3	26
SR15	0.54±0.16	20.3±5.8	0.91±0.24	0.96±0.29	0.13±0.06	3.7±1.5	0.42±0.31	26.9±6.1	35
SR16	0.25±0.08	5.2±1.6	0.34±0.09	0.38±0.12	0.05±0.02	1.17±0.48	0.38±0.38	7.8±1.8	12
SR17	0.12±0.04	9.5±2.8	0.56±0.16	0.24±0.07	0.04±0.02	2.22±0.90	1.25±0.98	14.0±3.1	19
SR18	0.52±0.16	20.5±5.9	0.84±0.22	44±12	1.8±1.1	11.7±4.8	4.8±1.6	84±15	117
SR19	0.20±0.06	3.8±1.1	0.24±0.06	8.2±2.3	0.83±0.61	3.3±1.3	1.66±0.84	18.2±3.3	26
SR20	0.34±0.11	9.5±2.7	0.48±0.12	23.5±6.5	0.01±0.01	5.3±2.2	1.34±0.66	40.4±7.6	34
SR21	0.36±0.10	1.75±0.51	0.17±0.05	1.52±0.48	0.01±0.01	0.89±0.36	0.23±0.10	4.92±0.88	7
SR22	0.28±0.09	7.0±2.0	0.20±0.05	32.3±9.1	–	6.3±2.6	0.87±0.34	46.9±9.9	50
SR23	0.34±0.10	1.68±0.48	0.12±0.03	2.02±0.59	–	1.16±0.46	0.50±0.50	5.8±1.2	10
Total	19.5±5.6	450±110	20.8±5.0	1110±280	29±12	310±160	145±41	2090±360	2536

**Table 15:** Event yields in off-${\text {Z}}$ ML regions. Yields shown as “–” have a contribution smaller than 0.01, or do not contribute to a particular region

	$\hbox {t}{\bar{\hbox {t}}} {\text {W}}$	$\hbox {t}{\bar{\hbox {t}}} {\text {Z}}$	$\hbox {t}{\bar{\hbox {t}}} {\text {H}}$	${\text {W}}$ ${\text {Z}}$	X+$\gamma$	Rare	Nonprompt lep.	SM expected	Data
SR1	8.8±2.5	11.2±3.2	7.0±1.8	87±24	18.8±9.5	22.0±9.0	68±25	222±36	285
SR2	0.15±0.07	0.15±0.08	0.10±0.03	1.23±0.50	0.02±0.02	0.43±0.22	0.61±0.61	2.7±1.7	2
SR3	2.38±0.69	2.07±0.59	1.60±0.42	13.6±3.8	0.26±0.11	4.2±1.7	11.3±4.9	35.5±6.4	34
SR4	0.18±0.06	0.14±0.04	0.08±0.02	0.24±0.11	–	0.22±0.12	0.13±0.13	0.99±0.31	2
SR5	1.11±0.31	1.64±0.47	1.02±0.26	11.2±3.1	0.89±0.84	1.68±0.68	4.6±2.0	22.1±4.0	29
SR6	0.91±0.25	0.64±0.19	0.59±0.15	4.7±1.4	0.05±0.02	1.05±0.44	1.76±0.80	9.7±1.7	8
SR7	26.8±7.5	29.7±8.5	21.9±5.7	7.0±2.0	6.9±3.3	11.3±4.7	113±41	217±44	272
SR8	7.9±2.3	5.0±1.5	4.6±1.2	1.14±0.36	0.87±0.37	2.43±0.99	15.7±5.6	37.7±6.8	56
SR9	3.8±1.1	5.3±1.5	3.47±0.89	0.82±0.26	0.86±0.40	1.91±0.78	5.2±2.5	21.4±3.7	21
SR10	2.69±0.78	1.78±0.52	1.76±0.45	0.35±0.12	0.27±0.11	1.07±0.45	2.9±1.4	10.9±1.9	18
SR11	22.1±6.4	19.5±5.7	16.1±4.3	0.44±0.13	6.4±2.6	5.1±2.1	19.4±6.6	89±14	112
SR12	5.0±1.5	2.99±0.86	2.93±0.79	0.09±0.03	0.64±0.27	1.28±0.53	2.67±0.94	15.6±2.4	20
SR13	3.22±0.96	4.2±1.2	3.21±0.84	0.12±0.05	0.56±0.24	1.56±0.64	3.5±1.7	16.4±2.7	23
SR14	1.53±0.45	1.31±0.38	1.23±0.32	–	0.16±0.07	0.77±0.32	0.36±0.36	5.36±0.95	7
SR15	0.91±0.28	2.00±0.60	1.94±0.54	0.03±0.01	0.26±0.11	2.6±1.1	1.27±0.83	9.0±1.6	12
SR16	4.6±1.4	4.6±1.3	3.30±0.87	5.4±1.5	1.9±1.1	3.4±1.4	5.3±1.7	28.4±3.9	46
SR17	0.13±0.06	0.18±0.07	0.05±0.02	0.19±0.19	–	0.16±0.11	–	0.72±0.41	2
SR18	3.7±1.1	2.24±0.67	2.42±0.66	3.8±1.1	0.15±0.07	3.1±1.3	2.4±1.1	17.8±2.8	25
SR19	0.25±0.09	0.18±0.05	0.07±0.02	0.10±0.03	–	0.26±0.12	0.04±0.04	0.89±0.29	0
SR20	3.4±1.0	1.71±0.50	1.59±0.42	4.2±1.2	0.12±0.05	3.1±1.3	3.6±2.0	17.7±3.3	31
SR21	0.28±0.09	0.21±0.06	0.07±0.02	0.16±0.07	–	0.36±0.15	0.12±0.12	1.20±0.32	2
Total	100±29	97±24	75±19	141±36	38±18	68±36	262±73	780±100	1007

**Table 16:** Event yields in LM regions. Yields shown as “–” have a contribution smaller than 0.01, or do not contribute to a particular region

	$\hbox {t}{\bar{\hbox {t}}} {\text {W}}$	$\hbox {t}{\bar{\hbox {t}}} {\text {Z}}$	$\hbox {t}{\bar{\hbox {t}}} {\text {H}}$	${\text {W}}$ ${\text {Z}}$	WW	X+$\gamma$	Rare	Charge misid.	Nonprompt lep.	SM expected	Data
SR1	7.7±2.1	2.86±0.84	2.22±0.57	56±15	53±15	38±19	12.5±5.3	13.4±1.5	51±36	235±47	309
SR2	1.69±0.50	1.06±0.30	1.12±0.28	1.99±0.56	1.80±0.53	3.5±2.3	1.14±0.53	0.70±0.08	6.3±4.3	19.3±5.2	26
SR3	23.0±6.4	8.6±2.5	7.1±1.9	5.9±1.7	5.5±1.6	14.8±7.7	6.1±2.6	6.97±0.79	64±37	142±39	156
SR4	6.3±1.8	3.15±0.89	4.4±1.1	0.34±0.09	0.25±0.10	1.22±0.57	1.96±0.81	0.77±0.09	13.8±8.2	32.2±8.8	38
SR5	17.6±5.0	6.1±1.7	5.6±1.5	0.48±0.14	0.37±0.12	4.2±2.1	2.6±1.1	4.71±0.54	11.4±5.8	53.0±9.1	69
SR6	6.0±1.7	3.17±0.91	4.2±1.1	0.10±0.04	0.12±0.04	1.05±0.45	2.00±0.83	0.68±0.08	4.8±3.0	22.0±4.0	30
SR7	2.37±0.71	1.19±0.36	1.42±0.40	0.08±0.03	–	0.61±0.36	2.08±0.86	0.53±0.06	1.8±1.4	10.1±2.0	21
SR8	0.09±0.05	0.01±0.01	0.02±0.01	0.23±0.10	0.80±0.22	–	0.04±0.02	0.06±0.01	0.27±0.27	1.53±0.48	3
SR9	0.36±0.11	0.17±0.06	0.11±0.03	0.12±0.04	0.11±0.04	0.17±0.10	0.19±0.08	0.03±0.00	0.32±0.30	1.58±0.41	0
SR10	0.16±0.08	0.04±0.03	0.01±0.01	0.25±0.17	0.77±0.27	1.3±1.3	0.10±0.10	0.06±0.02	0.16±0.16	2.9±2.9	1
SR11	0.44±0.18	0.02±0.02	0.08±0.03	–	0.13±0.08	0.01±0.01	0.16±0.07	0.05±0.02	0.41±0.41	1.31±0.93	4
Total	66±19	26.3±6.6	26.2±6.5	65±17	62±18	65±32	29±16	27.9±3.0	154±89	520±110	657

## Appendix B: Top five SRs for several representative models

Table 17 presents the top five SRs for several representative models, ranked based on the largest values of $N_\text {sig.}/\sqrt{\smash [b]{N_\text {bkg.} + N_\text {sig.}}}$, where $N_\text {sig.}$ and $N_\text {bkg.}$ are the signal and total background yields in each SR, respectively.

**Table 17:** Top five SRs for several representative models, ranked based on the largest values of $N_\text {sig.}/\sqrt{\smash [b]{N_\text {bkg.} + N_\text {sig.}}}$, where $N_\text {sig.}$ and $N_\text {bkg.}$ are the signal and total background yields in each SR, respectively

model	mass point	top SRs
$\mathrm {T}1{{\text {t}}{\text {t}}{\text {t}}{\text {t}}}$	$m_{{\tilde{\text {g}}}}=1400, m_{{\tilde{\chi }}^{0}_{1}}=400$	off-Z ML21, HH53, HH52, HH51, HH50
$\mathrm {T}1{{\text {t}}{\text {t}}{\text {t}}{\text {t}}}$	$m_{{\tilde{\text {g}}}}=2000, m_{{\tilde{\chi }}^{0}_{1}}=100$	HH53, HH52, off-Z ML21, HL39, HH49
$\mathrm {T}1{{\text {t}}{\text {t}}{\text {t}}{\text {t}}}$	$m_{{\tilde{\text {g}}}}=1800, m_{{\tilde{\chi }}^{0}_{1}}=100$	HH53, off-Z ML21, HH52, HL39, HH51
$\mathrm {T}1{{\text {t}}{\text {t}}{\text {t}}{\text {t}}}$	$m_{{\tilde{\text {g}}}}=1800, m_{{\tilde{\chi }}^{0}_{1}}=1000$	off-Z ML21, HH53, HH52, HH51, HH50
$\mathrm {T}1{{\text {t}}{\text {t}}{\text {t}}{\text {t}}}$	$m_{{\tilde{\text {g}}}}=1800, m_{{\tilde{\chi }}^{0}_{1}}=1550$	HH53, HL39, off-Z ML21, HH49, HH52
$\mathrm {T}6{{\text {ttWW}}}$	$m_{{\tilde{\text {b}}} _{1}}=1000, m_{{\tilde{\chi }}^\pm _{1}}=600$	off-Z ML21, HH53, HH51, HH50, HH52
$\mathrm {T}6{{\text {ttWW}}}$	$m_{{\tilde{\text {b}}} _{1}}=900, m_{{\tilde{\chi }}^\pm _{1}}=400$	off-Z ML21, HH51, HH50, HH53, off-Z ML20
$\mathrm {T}6{{\text {ttWW}}}$	$m_{{\tilde{\text {b}}} _{1}}=800, m_{{\tilde{\chi }}^\pm _{1}}=400$	off-Z ML21, HH51, HH50, HH34, off-Z ML20
$\mathrm {T}5{{\text {qqqqWZ}}}$	$m_{{\tilde{\text {g}}}}=1400, m_{{\tilde{\chi }}^{0}_{1}}=1$	on-Z ML23, HH53, HH52, HH51, HH49
$\mathrm {T}5{{\text {qqqqWZ}}}$	$m_{{\tilde{\text {g}}}}=900, m_{{\tilde{\chi }}^{0}_{1}}=600$	on-Z ML4, HH3, HH10, on-Z ML23, HH4
$\mathrm {T}5{{\text {qqqqWW}}}$	$m_{{\tilde{\text {g}}}}=1400, m_{{\tilde{\chi }}^{0}_{1}}=1$	HH53, HH52, HH49, HH51, HH50
$\mathrm {T}5{{\text {qqqqWW}}}$	$m_{{\tilde{\text {g}}}}=900, m_{{\tilde{\chi }}^{0}_{1}}=600$	HH3, HH10, HH4, HH7, HH50
$\mathrm {T}5{{\text {qqqqWZ}}}$ ($m_{{\tilde{\chi }}^\pm _{1}} = m_{{\tilde{\chi }}^{0}_{1}} + 20\,{\text {GeV}}$)	$m_{{\tilde{\text {g}}}}=1400, m_{{\tilde{\chi }}^{0}_{1}}=1$	HH59, HH53, HH52, HH62, HH51
$\mathrm {T}5{{\text {qqqqWZ}}}$ ($m_{{\tilde{\chi }}^\pm _{1}} = m_{{\tilde{\chi }}^{0}_{1}} + 20\,{\text {GeV}}$)	$m_{{\tilde{\text {g}}}}=900, m_{{\tilde{\chi }}^{0}_{1}}=600$	LL2, LL1, LL4, HL39, HL37
$\mathrm {T}5{{\text {qqqqWW}}}$ ($m_{{\tilde{\chi }}^\pm _{1}} = m_{{\tilde{\chi }}^{0}_{1}} + 20\,{\text {GeV}}$)	$m_{{\tilde{\text {g}}}}=1400, m_{{\tilde{\chi }}^{0}_{1}}=1$	HH59, HH53, HH52, HH51, HH62
$\mathrm {T}5{{\text {qqqqWW}}}$ ($m_{{\tilde{\chi }}^\pm _{1}} = m_{{\tilde{\chi }}^{0}_{1}} + 20\,{\text {GeV}}$)	$m_{{\tilde{\text {g}}}}=900, m_{{\tilde{\chi }}^{0}_{1}}=600$	LL2, LL4, HL39, LL1, HL37
$\mathrm {T}6{{\text {ttHZ}}}$ (${\mathcal {B}}({\tilde{\text {t}}} _{2}\rightarrow {\tilde{\text {t}}} _{1}{\text {Z}})$=1)	$m_{{\tilde{\text {t}}} _{2}}=850, m_{{\tilde{\text {t}}} _{1}}=625$	on-Z ML23, on-Z ML21, on-Z ML16, on-Z ML14, on-Z ML17
$\mathrm {T}6{{\text {ttHZ}}}$ (${\mathcal {B}}({\tilde{\text {t}}} _{2}\rightarrow {\tilde{\text {t}}} _{1}{\text {Z}})$=0.5)	$m_{{\tilde{\text {t}}} _{2}}=850, m_{{\tilde{\text {t}}} _{1}}=625$	on-Z ML17, on-Z ML23, on-Z ML21, on-Z ML14, on-Z ML16
$\mathrm {T}6{{\text {ttHZ}}}$ (${\mathcal {B}}({\tilde{\text {t}}} _{2}\rightarrow {\tilde{\text {t}}} _{1}{\text {Z}})$=0)	$m_{{\tilde{\text {t}}} _{2}}=850, m_{{\tilde{\text {t}}} _{1}}=625$	off-Z ML15, HH40, HH39, HH45, HH44
$\mathrm {T}1{{\text {qqqqL}}}$	$m_{{\tilde{\text {g}}}}=1600$	HH62, LM11, HH59, HH61, HH51
$\mathrm {T}1{{\text {qqqqL}}}$	$m_{{\tilde{\text {g}}}}=2400$	HH62, LM11, HH59, HH53, HH52
$\mathrm {T}1{{\text {tbs}}}$	$m_{{\tilde{\text {g}}}}=1200$	HH62, HH50, HH59, HH61, HH58
$\mathrm {T}1{{\text {tbs}}}$	$m_{{\tilde{\text {g}}}}=1700$	HH62, HH59, HH50, HH52, LM11
